# A Microbiota‐ and IL‐15‐Dependent Innate‐Like B Cell Progenitor Expressing E4BP4

**DOI:** 10.1002/advs.202512444

**Published:** 2025-12-02

**Authors:** Junming He, Xinlei Hou, Xiaomei Feng, Yayun Dong, Mengqi Ren, Surong Deng, Xinru Yang, Donglin Chen, Lingna Zhao, Shasha Chen, Meixiang Yang, Zhongjun Dong

**Affiliations:** ^1^ Department of Allergy the First Affiliated Hospital of Anhui Medical University and Institute of Clinical Immunology Anhui Medical University Hefei 230032 China; ^2^ Innovative Institute of Tumor Immunity and Medicine (ITIM) Hefei 230032 China; ^3^ Anhui Province Key Laboratory of Tumor Immune Microenvironment and Immunotherapy Hefei 230032 China; ^4^ Inflammation and Immune Mediated Diseases Laboratory of Anhui Province Anhui Medical University Hefei 230032 China; ^5^ State Key Laboratory of Membrane Biology School of Medicine and Institute for Immunology Tsinghua University Beijing 100084 China; ^6^ The Biomedical Translational Research Institute Guangzhou Key Laboratory for Germ‐Free Animals and Microbiota Application Key Laboratory of Ministry of Education for Viral Pathogenesis & Infection Prevention and Control School of Medicine Jinan University Guangzhou 510632 China; ^7^ Beijing Tsinghua Changgung Hospital School of Clinical Medicine Tsinghua University Beijing 102218 China; ^8^ Institute for Organ Transplant and Bionic Medicine Tsinghua University Beijing 102218 China

**Keywords:** B cell, NK‐B, NK cell, IL‐15 and E4BP4, PDK1

## Abstract

While natural killer (NK) cells and B cells arise from common lymphoid progenitors, the existence and nature of cells co‐expressing markers of both lineages (NK‐B cells) remain controversial. Here, this work identifies a significant population of CD3^−^NKp46^−^CD19⁺NK1.1⁺ cells, termed NK‐B cells, enriched in phosphoinositide‐dependent protein kinase‐1(PDK1)‐deficient mice but also present in wild‐type bone marrow. Single‐cell RNA sequencing and high‐dimensional flow cytometry reveal these NK‐B cells reside within early B cell developmental stages (pro‐B/pre‐B). Functionally, upon adoptive transfer into lymphocyte‐deficient hosts, NK‐B cells preferentially differentiated into B cells. Unlike conventional B cell precursors, NK‐B cells exhibit innate characteristics, including the capacity to secrete interferon‐gamma (IFN‐γ) and transforming growth factor‐beta (TGF‐β), and expressed CD122 (IL‐2/15Rβ) and Toll‐like receptor 9 (TLR9). Using a novel E4 promoter‐binding protein 4 (E4BP4) reporter model, this work demonstrates that both interleukin‐15 (IL‐15) signaling (via CD122) and TLR9‐mediated sensing of the gut microbiota cooperatively sustain E4BP4 expression within these progenitors. Consequently, germ‐free mice and mice deficient in IL‐15 or E4BP4 exhibit a profound loss of NK‐B cells. These findings unveil a distinct E4BP4‐expressing, innate‐like B cell progenitor pathway regulated by microbiota and IL‐15.

## Graphical abstract

This work identifies CD3.NKp46.CD19.NK1.1. NK‐B cells enriched in PDK1‐deficient yet present in wild‐type bone marrow. IL‐15 (via CD122) and TLR9‐microbiota signaling maintain their E4BP4 expression. These cells demonstrate innate immunity, preferentially differentiate into B cells, and unveil a novel microbiota‐ and IL‐15‐dependent regulatory pathway in lymphopoiesis.

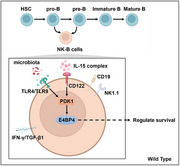

## 1 Introduction

Innate lymphocytes, such as natural killer (NK) cells, and adaptive lymphocytes, including B cells and T cells, both originate from common lymphoid progenitors.^[^ ^1^ ^]^ NK cells and B cells undergo development in the bone marrow and then migrate to the spleen to acquire their respective functions.^[^ ^2^, ^3^ ^]^ Specific markers are expressed at different stages during immune cell development. In the case of NK cell development, CD122 is expressed at the NKp stage, NK1.1 and NKp46 are expressed at the immature NK stage, and DX5 is expressed at the mature NK stage.^[^ ^4^ ^]^ B220 and CD19 expression are characteristic of B cell lineage.^[^ ^3^ ^]^ Although NK cells and B cells develop in similar anatomical sites, they have distinct roles in the immune system to defend against attacks. NK cells primarily exert protective immune responses through the coordination of cytotoxicity and cytokine secretion.^[^ ^5^, ^6^ ^]^ On the other hand, B cells secrete antibodies to induce neutralizing reactions with antigens.^[^ ^7^ ^]^ The existence of intertwined NK‐B cells, which share characteristics of both B cells and NK cells, is an intriguing question that warrants further exploration.

In the past, Lin^−^CD34^+^CD38^+^CD45RA^+^CD10^+^CD90^−^ cells isolated from humans were generally believed to be common progenitors of B cells and NK cells, capable of developing into both lineages.^[^ ^8^, ^9^, ^10^ ^]^ However, a recent discovery identified a new subset of lymphocytes called “natural killer‐like B cells” with the signature CD3^−^CD19^+^NK1.1^+^NKp46^+^. These cells are thought to originate from bone marrow pro‐B cells and have been found in the spleen and mesenteric lymph nodes. Natural killer‐like B cells possess unique characteristics distinct from conventional B cells and play a critical role in eliminating microbial infections through the secretion of interleukin‐18 (IL‐18) and interleukin‐12 (IL‐12). However, they lack the ability to produce IFN‐γ.^[^ ^11^ ^]^ Some researchers have suggested that the presence of these cells may be due to non‐specific binding of NK1.1 and NKp46 antibodies, and that this subset actually displays the phenotypic and functional characteristics of conventional B cells.^[^ ^12^ ^]^ Other studies have also found the existence of CD3^−^CD19^+^NKp46^+^ “NK‐B cells” in rhesus macaques and humans, which do have the ability to produce IFN‐γ, distinguishing them from NK‐B cells in mice.^[^ ^13^ ^]^ These findings indicate that while “NK‐B” cells exist not only in mice but also in rhesus macaques and humans, their characteristics differ among these biological models. Thus, the diverse attributes of “NK‐B” cells highlight the need for further comprehensive investigation. In this study, we discovered a significant population of NK‐like B cell progenitors in mice lacking phosphoinositide‐dependent kinase‐1 (PDK1).

PDK1, a member of the PI3K‐Akt signaling pathway, plays a critical role in the reconstitution ability of hematopoietic stem cells (HSCs).^[^ ^14^ ^]^ It is also essential for B cell development by regulating B cell Receptor (BCR) signaling. During the early stages of B cell development, PDK1 is involved in pre‐BCR conformation and can participate in interleukin‐7 signaling, influencing B cell survival and maturation.^[^ ^15^ ^]^ Moreover, PDK1 has an impact on B cell VDJ recombination, which is important for generating diverse antibody repertoires.^[^ ^16^, ^17^ ^]^ In the case of NK cells, PDK1 plays a crucial role in NK cell development by inducing E4BP4, a major transcription factor involved in NK cell development.^[^ ^18^, ^19^ ^]^

We utilized a mouse model lacking PDK1 and performed single‐cell RNA sequencing (scRNA‐seq) analysis to investigate the presence of a distinct subset of CD3^−^NKp46^−^CD19^+^NK1.1^+^ cells, which we refer to as NK‐B cells. However, these cells exhibit differences compared to the previously identified “natural killer‐like B cells” due to their lower or absent expression of NKp46. These NK‐B cells were found to be progenitors of B cells but possessed innate cell‐like functions, such as the secretion of IFN‐γ and TGF‐β1. We observed that their survival could be influenced by the IL‐15‐E4BP4 pathway and the microbiota. Our findings contribute to a better understanding of the unique progenitor status of B cells during their development.

## 2 Results

### 2.1 Discovery of CD19⁺NK1.1⁺ NK‐B Cells in PDK1‐Deficient Mice

PDK1 deficiency has been shown to cause severe developmental defects in T and B cells.^[^ ^14^, ^15^, ^18^, ^19^ ^]^ In a previous study, we investigated the role of PDK1 in NK cell development using a mouse model called PDK1^fl/fl^/Vav1‐Cre^+^.^[^ ^19^ ^]^ Our findings revealed that the absence of PDK1 led to an increased population of NK1.1^+^ NK cells with reduced expression of CD122, a subunit of the IL‐15 receptor. We hypothesized that PDK1 is involved in regulating IL‐15‐induced E4bp4, which determines the expression of CD122. However, we cannot exclude the possibility that this CD3^−^CD122^−/low^ NK1.1^+^ cell population represents a novel subset of lymphocytes that develop abnormally in PDK1‐deficient mice.

**Figure 1:**
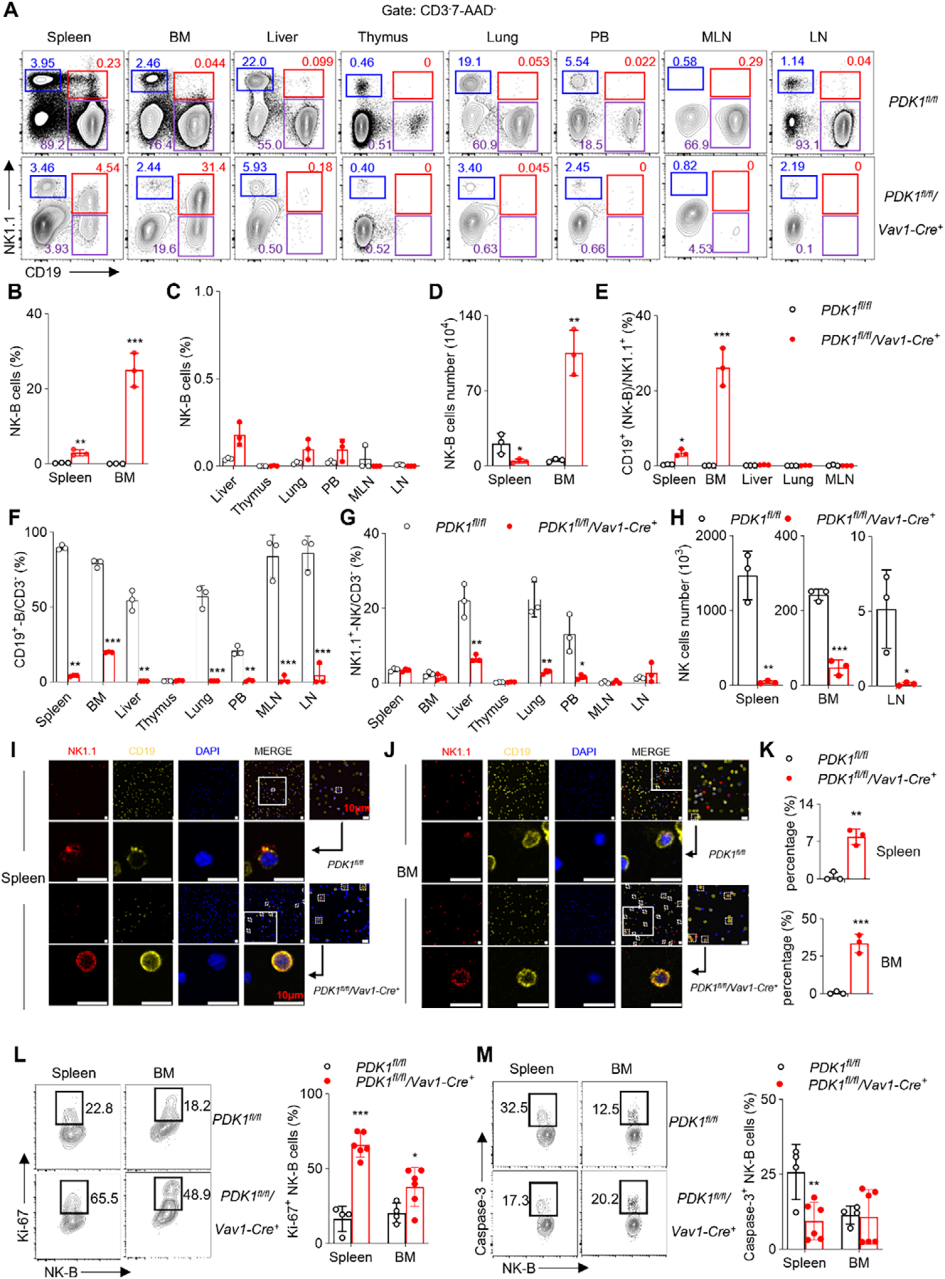
Discovery of Novel Mouse NK‐B Cells Expressing CD19 and NK1.1. A) Lymphocytes in different mouse tissues were isolated, stained with antibodies against CD3, CD19 and NK1.1 and analyzed by flow cytometry. Dead cells were excluded by 7‐AAD staining. CD3^−^7‐AAD^−^ lymphocytes were gated out for analyzing NK1.1 versus CD19 and representative plots were shown. B,C) Percentages and D) absolute numbers of CD3^−^7‐AAD^−^CD19^+^NK1.1^+^ cells referring to as “NK‐B” were quantified (n = 3). BM, bone marrow; MLN, mesenteric lymph nodes; LN, inguinal lymph nodes; PB, peripheral blood. E) Percentages of CD3^−^CD19^+^ cells/NK1.1^+^ cells were calculated (n = 3). Percentages of F) CD3^−^NK1.1^−^CD19^+^ B cells and G) CD3^−^NK1.1^+^CD19^−^ NK cells were calculated (n = 3). Absolute number of CD3^−^NK1.1^+^CD19^−^ NK cells H) were calculated (n = 3). Immunofluorescence staining of NK‐B cells in the I) spleen and J) bone marrow of the indicated mice, percentage K) in field of same size was also shown (n = 3). Red indicated NK1.1, yellow indicated CD19 and blue indicated DAPI. Scale bar represents 10 µm. Lymphocytes in different mouse tissues were isolated, stained with antibodies against L) CD3, CD19, NK1.1, Ki‐67 or M) Caspase‐3 and analyzed by flow cytometry (n = 6). CD3^−^CD19^+^NK1.1^+^ NK‐B cells were gated out for analyzing Ki‐67 or Caspase‐3. Data represent the mean ± s.d. are representative of at least three independent experiments. *p < 0.05, **p < 0.01 and ***p < 0.001. Unpaired Student's *t*‐tests (two‐tailed) was used to calculate these values.

In this study, we aimed to identify the lymphocyte population observed in PDK1^fl/fl^/Vav1‐Cre^+^ mice. Initially, immune cells were gated to exclude doublets and dead cells, and the CD3^−^ cell population was analyzed (Figure S1A, Supporting Information). Interestingly, we found a significant increase in the percentage of CD3^−^NK1.1^+^ NK cells expressing the B cell‐specific marker CD19 in the spleen and bone marrow of PDK1^fl/fl^/Vav1‐Cre^+^ mice compared to wild‐type control mice. The increase was ≈20‐fold in the spleen and 500‐fold in the bone marrow (**Figure** **1**A,B). However, these cells were almost undetectable in other organs including the liver, thymus, lung, peripheral blood (PB), mesenteric lymph nodes (MLNs), and inguinal lymph nodes (LN) (Figure 1A,C).

Moreover, the absolute number of CD3^−^CD19^+^NK1.1^+^ cells in the bone marrow showed a more than 50‐fold increase compared to wild‐type mice (Figure 1D). To provide a specific name for this newly identified subset of lymphocytes, we termed them “NK‐B cells” (Figure S1A, Supporting Information). We further validated these results by gating on NK1.1^+^ cells and subsequently analyzing the presence of CD3 and CD19 markers (Figure S1B, Supporting Information). Consistently, our results confirmed that the deletion of PDK1 significantly increased the percentage of CD3^−^CD19^+^ cells within the NK1.1^+^ population in the spleen and bone marrow. This increase was even greater in the bone marrow, exceeding a 100‐fold (Figure 1E; Figure S1C, Supporting Information).

In contrast to the NK‐B cells, the percentage and number of B cells (CD3^−^CD19^+^NK1.1^−^) and NK cells (CD3^−^CD19^−^NK1.1^+^) decreased in the detected organs of PDK1^fl/fl^/Vav1‐Cre^+^ mice compared to wild‐type controls as expected (Figure 1A,F–H; Figure S1C, Supporting Information).

Immunofluorescence staining further confirmed that the percentage of NK‐B cells was higher in both the spleen (Figure 1I) and bone marrow (Figure 1J) of PDK1^fl/fl^/Vav1‐Cre^+^ mice compared to wild‐type mice (Figure 1K). Flow cytometry further revealed that we tested those cells for proliferation and apoptosis compared to wild‐type mice. We could see NK‐B cells in PDK1^fl/fl^/Vav1‐Cre^+^ mice showed increased expression of Ki‐67 as a marker indicating proliferating cells (Figure 1L), while the expression of Caspase‐3 was reduced in spleen (Figure 1M).

### 2.2 Identification of NK‐B Cell Subpopulation that Lacks NKp46 Expression

**Figure 2:**
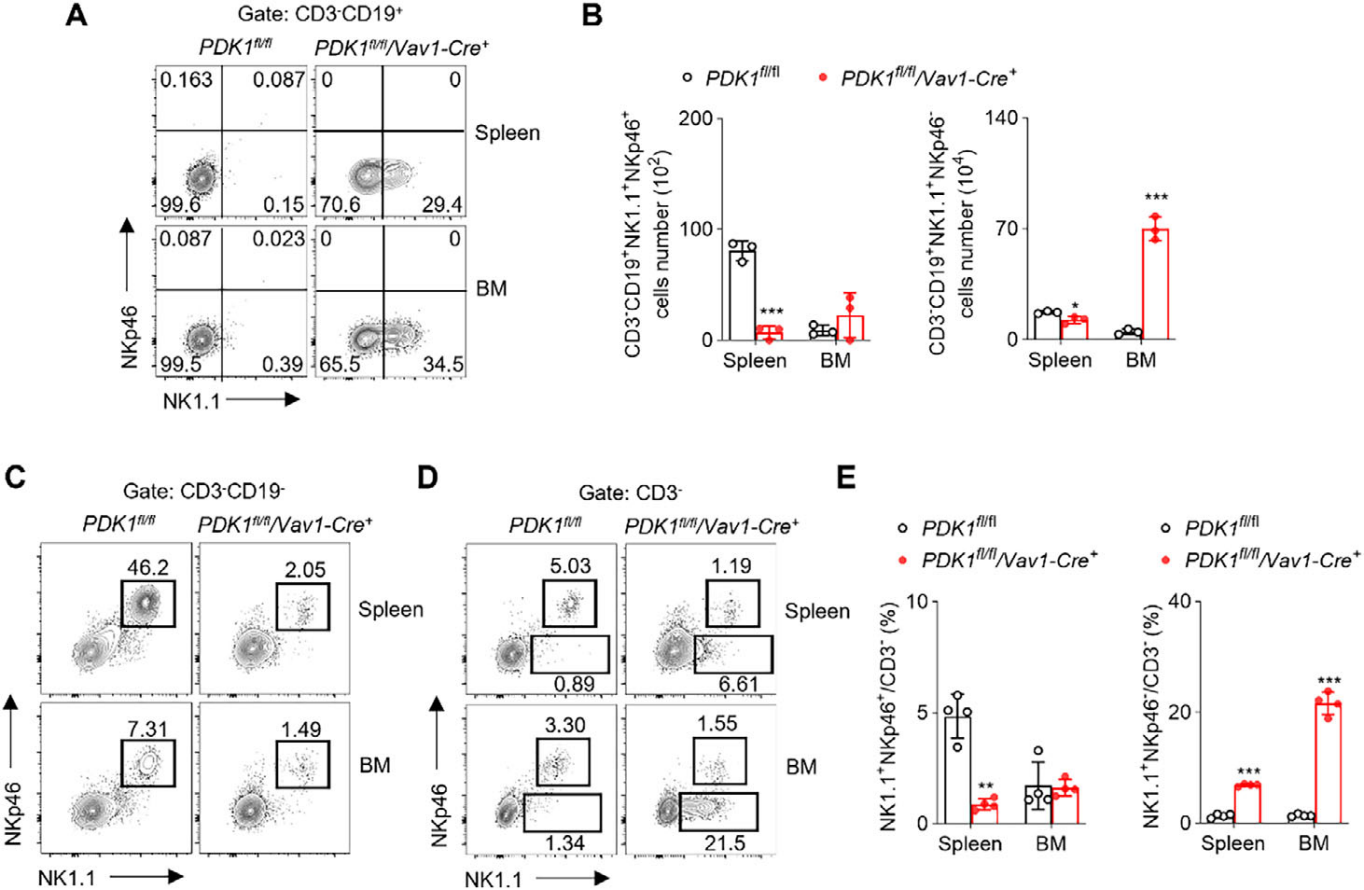
Identification of NK‐B Cell Subpopulation that Lacks NKp46 Expression. A) Lymphocytes in different mouse tissues were isolated, stained with antibodies against CD3, CD19, NK1.1 and NKp46 and analyzed by flow cytometry. CD3^−^CD19^+^ lymphocytes were gated out for analyzing NK1.1 versus NKp46. B) Absolute number of CD3^−^CD19^+^NK1.1^+^NKp46^−^ cells and CD3^−^CD19^+^NK1.1^+^NKp46^+^ cells were calculated (n = 3). C) Lymphocytes in different mouse tissues were isolated, stained with antibodies against CD3, CD19, NK1.1 and NKp46 and analyzed by flow cytometry. CD3^−^CD19^−^ lymphocytes were gated out for analyzing NK1.1 versus NKp46. D) Lymphocytes in different mouse tissues were isolated, stained with antibodies against CD3, NK1.1 and NKp46 and analyzed by flow cytometry. CD3^−^ lymphocytes were gated out for analyzing NK1.1 versus NKp46. E) Percentage of NK1.1^+^NKp46^+^/CD3^−^ cells and NK1.1^+^NKp46^−^/CD3^−^ cells were calculated (n = 4). Data represent the mean ± s.d. are representative of at least three independent experiments. *p < 0.05, **p < 0.01 and ***p < 0.001. Unpaired Student's *t*‐tests (two‐tailed) was used to calculate these values.

NKp46 serves as a marker for identifying NK‐B cells, as previously reported by Wang et al.^[^ ^11^ ^]^ Surprisingly, we observed that the majority of CD3^−^CD19^+^NK1.1^+^ cells in PDK1^fl/fl^/Vav1‐Cre^+^ mice did not express NKp46 (**Figure** **2**A). We noted an increase in the number of CD3^−^CD19^+^NK1.1^+^NKp46^−^ cells in the bone marrow, while the CD3^−^CD19^+^NK1.1^+^NKp46^+^ cells significantly decreased in the spleen (Figure 2B). Interestingly, when analyzing the CD3^−^CD19^−^ population, we did not observe the emergence of the NK1.1^+^NKp46^−^ subset, unlike what was observed in the CD3^−^CD19^+^ population (Figure 2C). Additionally, by examining the CD3^−^ population, we found a decrease in NK1.1^+^NKp46^+^ cells and an increase in NK1.1^+^NKp46^−^ cells in PDK1^fl/fl^/Vav1‐Cre^+^ mice (Figure 2D,E). In summary, our findings demonstrate that NK‐B cells in PDK1^fl/fl^/Vav1‐Cre^+^ mice are predominantly characterized by a lack of NKp46 expression. This is in contrast to the previously described “natural killer‐like B cells” which exhibit consistent and high levels of NKp46 in the spleen and mesenteric lymph nodes,^[^ ^11^ ^]^ suggesting the existence of a novel subset within the CD3^−^CD19^+^NK1.1^+^ cell population.

### 2.3 Discovery of NK‐B Cell Population through Single‐Cell RNA Sequencing

**Figure 3:**
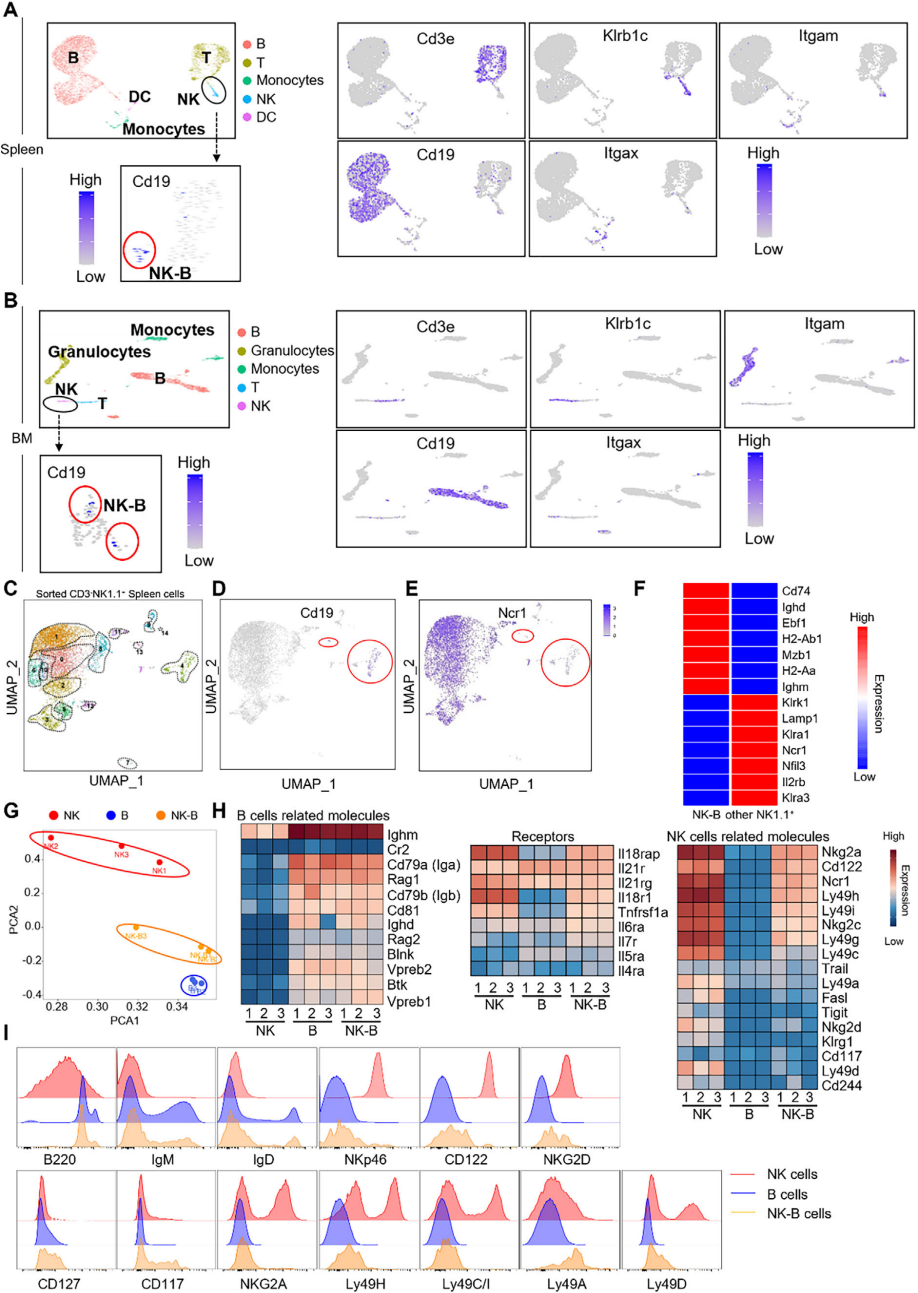
NK‐B Cells Have Unique Signature Features. UMAP clustering plot of A) wild‐type spleen cells and B) bone marrow cells from *Tabula Muris* database. Each point represents an individual cell. Cell types were determined by differential gene expression of known markers between clusters. NK‐B cells were determined by CD3^−^NK1.1^+^CD19^+^ gene expression. The color key indicates the expression level. C) UMAP clustering plot of 10000 wild‐type mice CD3^−^NK1.1^+^ spleen cells. Each point represents an individual cell. Cells were grouped into 14 distinct clusters. D) Distribution of Cd19 gene expression in UMAP plot. The color key indicates the expression level. E) Distribution of Ncr1 gene expression in UMAP plot. The color key indicates the expression level. F) Comparison of NK‐B cells and other NK1.1^+^ cells in scRNA‐Seq populations. Heat map illustrating different gene expression in NK‐B cells and other NK1.1^+^ cells. The color key indicates the expression level. G) CD3^−^NK1.1^+^CD19^+^ NK‐B cells, CD3^−^NK1.1^−^CD19^+^ B cells and CD3^−^NK1.1^+^CD19^−^ NK cells from wild‐type mice bone marrow were isolated by flow cytometer. PCA analyze showing the relationship between those three types cells in RNA‐Seq. H) Heat map illustrating the transcript expression of the indicated genes. Each panel represents an individual mouse. The color key indicates the expression level. I) Flow cytometry analysis of expression of the indicated receptors on bone marrow CD3^−^NK1.1^+^CD19^−^ NK cells (red line), CD3^−^NK1.1^−^CD19^+^ B cells (blue line) and CD3^−^NK1.1^+^CD19^+^ NK‐B cells (orange line) from wild‐type mice.

We performed additional analysis on scRNA‐Seq data obtained from the *Tabula Muris* database, specifically from the wild‐type mice spleen (GSE109774) and bone marrow (GSE109774). This analysis revealed the existence of a unique subset of cells expressing CD19 mRNA within the NK1.1(*Klrb1c*) positive population (**Figure** **3**A,B). However, due to their rarity in wild‐type mice, it was challenging to conduct a comprehensive analysis using the available databases mentioned above. To overcome this limitation, we isolated CD3^−^NK1.1^+^ cells from the bone marrow of wild‐type mice and performed scRNA‐Seq analysis. Through examination of the variations in molecule expression, we further classified CD3^−^NK1.1^+^ cells into 14 distinct subpopulations (Figure 3C). Interestingly, clusters 4 and 13 exhibited *Cd19* expression but lacked the *Ncr1* encoding NKp46, suggesting that they may represent the newly identified NK‐B cells (Figure 3D,E). Gene profiling analysis revealed a unique gene expression pattern in NK‐B cells, setting them apart from conventional NK (other NK1.1^+^) cells. To further investigate their developmental origin and identity, we performed fate‐mapping experiments using R26^stop^YFP/Ncr1‐Cre^+^ reporter mice, in which the activity of the Cre enzyme is monitored by the expression of YFP (*Ncr1*). Consistent with our previous scRNA‐seq results, fate‐mapping analysis in the spleen and bone marrow demonstrated significantly lower YFP signal in NK‐B cells compared to conventional NK cells, confirming absent or very low expression of *Ncr1* (Figure S2A,B, Supporting Information). Furthermore, NK‐B cells exhibited high expression levels of B cell‐related genes *Ighm* and *Ighd* (Figure 3F). Therefore, the scRNA‐Seq data unambiguously demonstrate the presence of the NK‐B cell population in the spleen and bone marrow of wild‐type mice, albeit with a remarkably low proportion. Moreover, this population displays its own distinct transcriptional profile. To investigate whether human bone marrow and spleen contain CD3^−^NKp46^−^CD19⁺ cells expressing NK1.1 homologs, we analyzed published single‐cell RNA‐seq datasets from human bone marrow (66 613 cells) and spleen (200 664 cells). This population showed no expression of key NK‐cell receptors, including activating markers (*Klrk1*/NKG2D, *Cd226*/DNAM‐1, *Ncr2*/NKp44, *Fcgr3a*/CD16), the inhibitory complex Klrc1/Klrd1 (NKG2A/CD94), the adhesion marker *Ncam1* (CD56), or *Klrb1* (CD161)‐an inhibitory C‐type lectin receptor commonly used to identify human NK cells. These results indicate that the CD3^−^CD19⁺NKp46^−^ population in both human bone marrow and spleen lacks a broad repertoire of NK‐cell characteristic receptors, confirming its phenotypic distinction from conventional NK‐cell lineages. (Figure S3A,B, Supporting Information). Thus, the presence of NK‐B cells in humans remains elusive.

### 2.4 Distinct Molecular Profile of NK‐B Cells

To further explore NK‐B characteristics, we analyzed the gene expression profiles of the sorted NK‐B cells (CD3^−^CD19^+^NK1.1^+^), B cells (CD3^−^CD19^+^NK1.1^−^), and NK cells (CD3^−^CD19^−^NK1.1^+^) in the bone marrow of wild‐type mice by RNA‐Seq. Principal component analysis (PCA) of RNA‐Seq data revealed that although the transcription profiles of NK‐B cells were closer to B cells, they were not completely overlapping, indicating that NK‐B cells represent a distinct cell population with unique features (Figure 3G).

Importantly, NK‐B cells expressed B cell‐specific genes associated with the B cell receptor (BCR) complex, such as *Cd79a (Iga)*, *Cd79b (Igb)*, *Ighm*, and *Ighd*, at levels comparable to B cells (Figure 3H). Additionally, NK‐B cells expressed certain receptors like *Il18r1* and *Il7r* (Figure 3H). They also exhibited lower expression of NK cell‐related molecules, including *CD122*, and *Ncr1*, compared to NK cells (Figure 3H). Flow cytometry analysis was conducted to confirm these observations (Figure 3I). Furthermore, NK‐B cells expressed some functional receptors of NK cells, including NKG2D, NKG2A, Ly49H, and Ly49C/I, albeit at lower levels than NK cells (Figure 3H,I). Notably, NK‐B cells exhibited high levels of CD117, an immature marker for immune cells, suggesting that NK‐B cells may represent a progenitor subset (Figure 3I).

Considering the expression of BCR IgM and IgD, we also examined the expression of rearrangement‐related genes in NK‐B cells. The RNA‐Seq data revealed substantial expression of the pre‐BCR complex components, including *Iga*, *Igb*, *Vpreb1*, and *Vpreb2*, in NK‐B cells (Figure 2H). These findings were further confirmed by quantitative Polymerase Chain Reaction (qPCR) analysis (Figure S4A, Supporting Information). Moreover, we observed the presence of both distal (*VHJ588*) and proximal (*VHJ7183*) VH (Heavy) regions germline transcripts in NK‐B cells. Additionally, germline transcripts of Vκ‐Jκ recombination, associated with Igκ (Light) chain, were detected in NK‐B cells (Figure S4A, Supporting Information). These results suggest that NK‐B cells may represent a population closely related to B cells.

Interestingly, the deletion of PDK1 resulted in upregulated expression of CD117 and CD127 in NK‐B cells, accompanied by downregulated expression of other molecules such as B220, CD122, IgM, IgD, and NKG2D (Figure S4B, Supporting Information). These findings suggest that the loss of PDK1 hinders the differentiation of NK‐B cells. Due to the altered expression patterns of molecules on NK‐B cells in PDK1‐deficient mice, it is plausible that NK‐B cells in PDK1^fl/fl^/Vav1‐Cre^+^ mice may differ from those in wild‐type mice.

### 2.5 NK‐B Cells: Unveiling Innate Cell‐Like Functions

**Figure 4:**
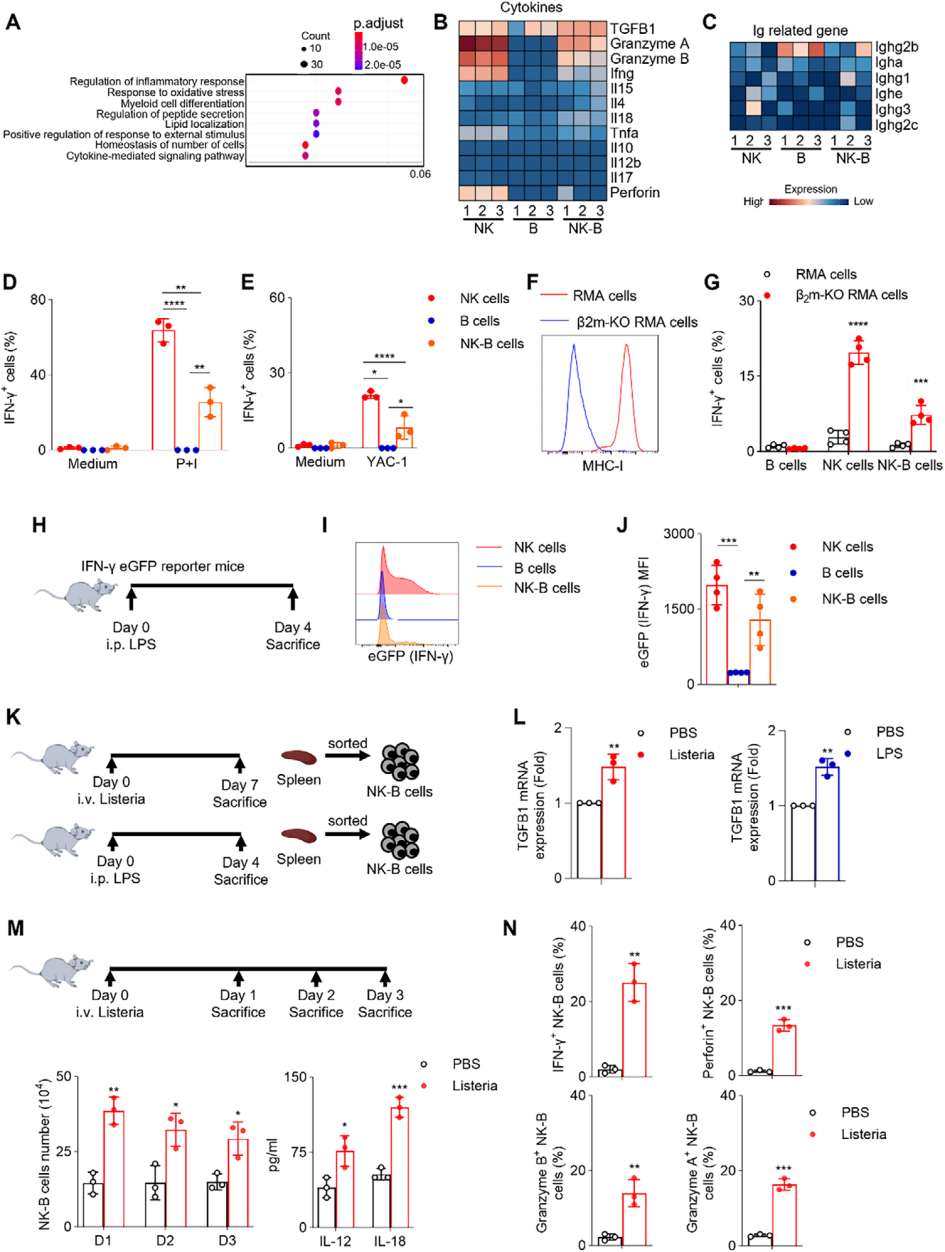
NK‐B Cells: Unveiling Innate Cell‐Like Functions. A) Gene expression up‐regulated in NK‐B cells (CD3^−^CD19^+^NK1.1^+^) compared with NK cells (CD3^−^CD19^−^NK1.1^+^) and B cells (CD3^−^CD19^+^NK1.1^−^) from RNA‐Seq were analyzed by using pathway analysis based on KEGG database. B,C) Heat map illustrating the average transcript expression of the indicated genes (n = 3). The color key indicates the expression level. Poly(I:C)‐primed splenocytes from the wild‐type mice were stimulated with the indicated stimuli, phorbol‐12‐myristate‐13‐acetate (PMA) D) plus ionomycin (P+I) and E) tumor target cells (n = 3). Medium served as the negative control. Percentages of IFN‐γ^+^ gated NK cells (CD3^−^CD19^−^NK1.1^+^), B cells (CD3^−^CD19^+^NK1.1^−^) and NK‐B cells (CD3^−^CD19^+^NK1.1^+^) were analyzed. F) Flow cytometry analysis of expression of MHC‐I on RMA cell line (red line) and β2m‐KO RMA cell line (blue line). G) Poly(I:C)‐primed splenocytes from the wild‐type mice were stimulated with the tumor target cells (n = 4). Percentages of IFN‐γ^+^ gated NK cells (CD3^−^CD19^−^NK1.1^+^), B cells (CD3^−^CD19^+^NK1.1^−^) and NK‐B cells (CD3^−^CD19^+^NK1.1^+^) were analyzed. H) IFN‐γ‐eGFP reporter mice were treated with LPS for 4 days. I) The eGFP expression level in indicated cells from spleen were shown. J) The absolute MFI (eGFP‐MFI) were quantified (n = 4). K) Experimental design of different in vivo wild‐type mice model. Wild‐type mice were treated with Listeria for 7 days (up), LPS for 4 days (down). PBS were used as control. L) TGF‐β1 expression fold change in CD3^−^NK1.1^+^CD19^+^ NK‐B cells in different priming model was analyzed using qPCR (n = 3). M) Wild‐type mice were infected with Listeria and analyzed at various time points. The percentage of NK‐B cells (CD3^−^CD19^+^NK1.1^+^) was determined. NK‐B cells were isolated on the specified days, cultured for 24 h, and subsequently subjected to ELISA to assess cytokine production (n = 3). N) Wild‐type mice were infected with Listeria and sacrificed at the indicated time points. Spleens were harvested, percentages of IFN‐γ^+^, Perforin^+^, Perforin A^+^, and Granzyme B^+^ gated NK‐B cells (CD3^−^CD19^+^NK1.1^+^) were analyzed (n = 3). Data represent the mean ± s.d. are representative of at least three independent experiments. *p < 0.05, **p < 0.01, ***p < 0.001 and ****p < 0.0001. Unpaired Student's t‐tests (two‐tailed) was used to calculate these values.

Functional enrichment analysis of the NK‐B cell transcriptome revealed an enrichment of inflammatory pathways, suggesting that NK‐B cells may participate in immune responses (**Figure** **4**A). Further analysis of RNA‐Seq data showed that NK‐B cells exhibit high expression levels of TGF‐β1, Granzyme, and IFN‐γ (Figure 4B). However, antibody‐related genes were almost non‐expressing in NK‐B cells (Figure 4C).

NK‐B cells produced IFN‐γ when stimulated with ionomycin combined with PMA, although at lower levels compared to NK cells (Figure 4D). Similarly, NK‐B cells also exhibited the ability to produce IFN‐γ when stimulated with YAC‐1 cells (Figure 4E). Since NK‐B cells express functional receptors similar to NK cells, we investigated whether MHC‐I‐dependent inhibitory signaling could suppress the activation of NK‐B cells. To investigate this, we generated MHC‐I‐deficient RMA cells, which trigger “missing‐self” recognition by NK cells (Figure 4F). Upon PolyI:C activation, NK‐B cells demonstrated an elevated expression of IFN‐γ when stimulated with β2m‐deficient RMA cells, while displaying a reduced response to RMA cells (Figure 4G). These findings suggest that NK‐B cells engage in innate recognition through a mechanism akin to that of NK cells, known as “missing‐self.”

Furthermore, we assessed the ability of NK‐B cells to produce IFN‐γ in vivo using eGFP‐IFN‐γ‐reporter mice. Following intraperitoneal injection of LPS in mice, both splenic NK cells and NK‐B cells were observed to produce IFN‐γ (Figure 4H–J), providing further evidence of the in vivo capacity of NK‐B cells to produce IFN‐γ.

Transforming growth factor‐beta (TGF‐β) is a versatile cytokine that plays a crucial role in regulating various immune cellular functions. To explore the ability of NK‐B cells to produce TGF‐β1, as indicated by RNA‐seq analysis, we conducted in vivo experiments (Figure 4K). The findings demonstrated that the administration of Listeria or LPS in wild‐type mice resulted in an upregulation of TGF‐β1 mRNA expression levels in NK‐B cells (Figure 4L), suggesting the potential involvement of NK‐B cells in the modulation of inflammation. To further elucidate the role of NK‐B cells during infection, we characterized their expansion dynamics in wild‐type mice following Listeria challenge. It was discovered that the splenic CFU in mice infected with Listeria bacteria gradually increased over time (Figure S5A–C, Supporting Information). A significant increase in the NK‐B cell number was observed on days 1–3 post‐infection. NK‐B cells isolated at day 1 and cultured for 24 h exhibited markedly elevated secretion of IL‐12 and IL‐18, demonstrating their functional activation in response to infection (Figure 4M; Figure S5D, Supporting Information). Although the expression levels of effector molecules in NK‐B cells were lower than those in conventional NK cells, they exhibited high proportions of IFN‐γ⁺, Perforin⁺, Granzyme A⁺, and Granzyme B⁺ populations following infection (Figure 4N; Figure S5E, Supporting Information), indicating their acquisition of cytotoxic function during immune challenge. To delineate the non‐redundant function of NK‐B cells in anti‐bacterial immunity, we utilized an adoptive transfer approach. Pre‐activated NK and NK‐B cells were isolated from wild‐type mice on day 3 post‐infection and transferred into immunodeficient Rag1^−^^−^γc^−^^−^ recipients, either individually or in combination at an 18:1 physiological ratio. Recipients were then challenged intravenously with 1000 CFU of Listeria, and splenic bacterial burden was evaluated 3 days post‐infection. Mice receiving NK‐B cells alone exhibited a significant reduction in bacterial load relative to unreconstituted controls, indicating their intrinsic ability to limit bacterial replication. Moreover, co‐transfer of NK and NK‐B cells led to a more substantial decrease in bacterial burden than did transfer of NK cells alone (Figure S5F, Supporting Information), highlighting a synergistic role of NK‐B cells in concert with conventional NK cells. Thus, our findings establish that NK‐B cells play a non‐redundant role through both intrinsic antibacterial functions and synergy with NK cells.

### 2.6 NK‐B Cells Represent a Subset of B‐Cell Progenitors

**Figure 5:**
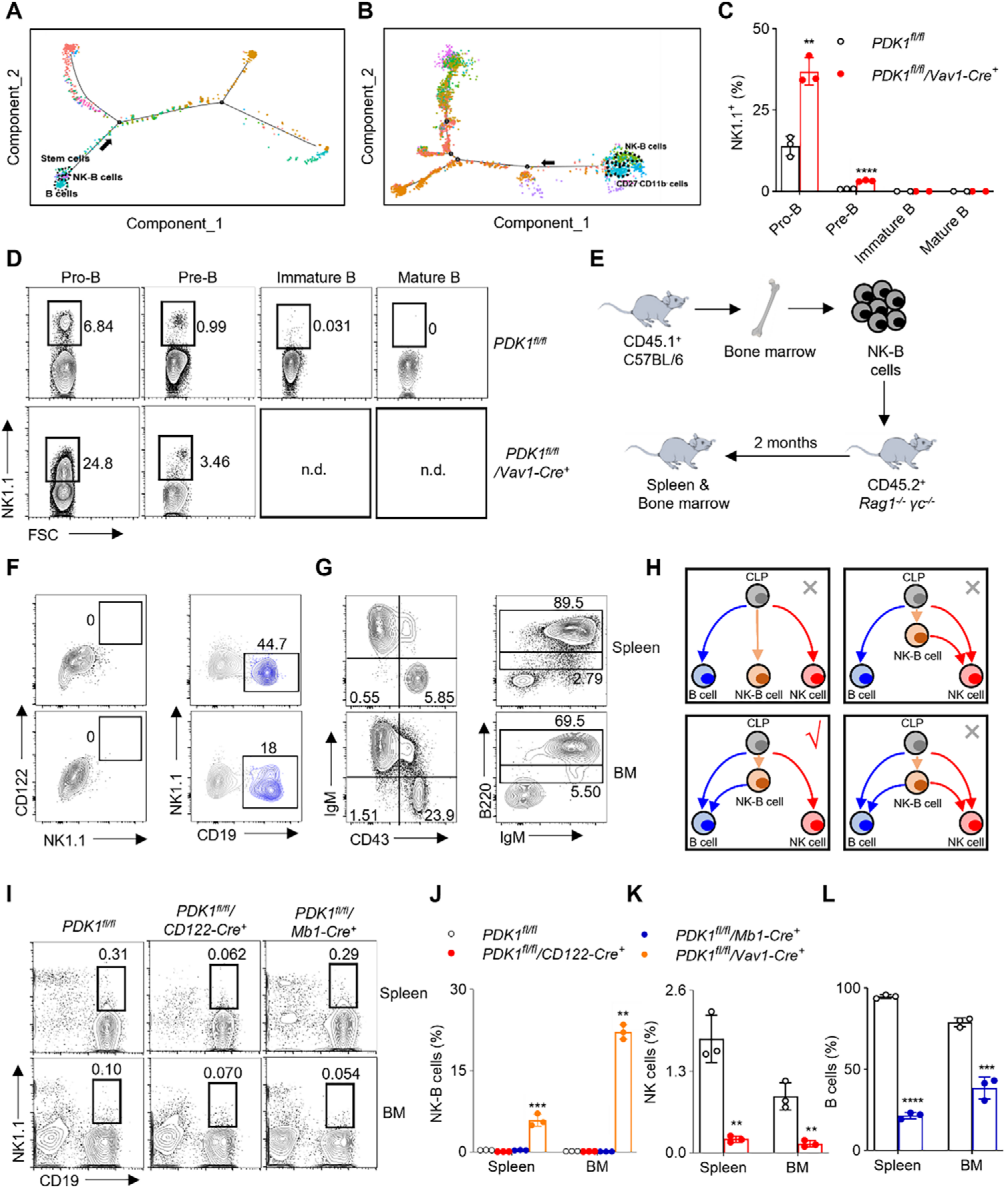
NK‐B Cells Represent a Subset of B‐Cell Progenitors. A) Monocle trajectories of wild‐type bone marrow lymphocytes from *Tabula Muris* database colored by cluster identity. Each dot represents a single cell. Cell orders are inferred from the expression of the most variable genes across all cells. The trajectory direction was determined by biological prior. Green dots indicated stem cells, pink dots indicated NK‐B cells and blue dots indicated B cells. B) Monocle trajectories of wild‐type bone marrow CD3^−^NK1.1^+^ cells colored by cluster identity. Each dot represents a single cell. Cell orders are inferred from the expression of the most variable genes across all cells. The trajectory direction was determined by biological prior. Green dots indicated NK‐B cells and blue dots indicated CD27^−^CD11b^−^ immature NK cells. C) Representative percentage and D) flow cytometric plots of NK1.1 expression in pro‐B cells (CD43^+^IgM^−^), pre‐B cells (CD43^−^IgM^−^), immature B cells (B220^int^IgM^+^) and mature B cells (B220^+^IgM^+^) in the bone marrow of the indicated mice (n = 3). E) Schematic representation. CD3^−^NK1.1^+^CD19^+^ NK‐B cells from CD45.1^+^ mice bone marrow were sorted and transferred into CD45.2^+^Rag1^−/−^γc^−/−^ mice and analyzed at 2 months. F) NK cells (CD3^−^CD19^−^NK1.1^+^CD122^+^), B cells (CD3^−^CD19^+^NK1.1^−^) and NK‐B cells (CD3^−^CD19^+^NK1.1^+^) existence was detected in recipient mice spleen and bone marrow. G) B cells development stage pro‐B cells (CD43^+^IgM^−^), pre‐B cells (CD43^−^IgM^−^), immature B cells (B220^int^IgM^+^) and mature B cells (B220^+^IgM^+^) existence was detected in recipient mice spleen and bone marrow (n = 3). H) Schemes depict four possible scenarios of NK‐B cell differentiation. Lymphocytes from indicated mice were isolated, stained with antibodies against CD3, CD19 and NK1.1 and analyzed by flow cytometry. CD3^−^ lymphocytes were gated out for analyzing NK1.1 versus CD19 I). Percentages of NK‐B cells (CD3^−^CD19^+^NK1.1^+^) J), NK cells (CD3^−^CD19^−^NK1.1^+^) K) and B cells (CD3^−^CD19^+^NK1.1^−^) L) were calculated (n = 3). Data represent the mean ± s.d. are representative of at least three independent experiments. *p < 0.05, **p < 0.01, ***p < 0.001 and ****p < 0.0001. Unpaired Student's *t*‐tests (two‐tailed) was used to calculate these values.

RNA velocity analysis of single‐cell differentiation trajectories using the *Tabula Muris* database revealed that the NK‐B cell cluster occupies a similar position in terms of differentiation activity as hematopoietic stem cells and B cells (**Figure** **5**A). Furthermore, based on our scRNA‐Seq data, the NK‐B cell cluster was shown to occupy a similar position as the immature CD27^−^CD11b^−^ cells (Figure 5B), indicating that NK‐B cells are in an immature stage. Principal Component Analysis (PCA) analysis demonstrated a closer relationship between NK‐B cells and B cells (Figure 3G), and cell trajectory analysis indicates that NK‐B cells may precede B cells, suggesting that NK‐B cells could potentially serve as progenitor cells in the development of B cells.

To verify this hypothesis, we first examined the expression of NK1.1 at different developmental stages of B cells. The results revealed that NK1.1 is expressed in the early stages of B cell development, specifically in the pro‐B (CD43^+^IgM^−^) and pre‐B (CD43^−^IgM^−^) stages (Figure 5C,D). However, NK1.1 expression was not detected in the late stages of B cell development, including immature B (B220^int^IgM^+^) and mature B (B220^+^IgM^+^) stages (Figure 5C,D). In PDK1^fl/fl^/Vav1‐Cre^+^ mice, a higher percentage of NK1.1 expression was observed in the pro‐B and pre‐B stages of bone marrow compared to wild‐type mice (Figure 5C,D).

Second, we transferred CD45.1^+^ NK‐B cells isolated from the bone marrow of wild‐type mice into CD45.2^+^ Rag1^−/−^γc^−/−^ mice (Figure 5E). After 2 months, analysis of the CD45.1^+^ cells in the recipient mice revealed the absence of NK cells and NK‐B cells, but the presence of B cells in the spleen and bone marrow (Figure 5F). Additionally, mature B cells were also detected in the spleen and bone marrow of recipient mice (Figure 5G). To determine whether B cells differentiated from NK‐B cells are functionally or phenotypically distinct from conventional B cells, we compared mature B cells derived from adoptively transferred NK‐B cells with wild‐type B cells. Flow cytometric analysis of CD38, CD138, and IL‐10 expression revealed no statistically significant differences between the two groups. These results indicate that NK‐B cell‐derived B cells are phenotypically and functionally comparable to conventional B cells, suggesting that NK‐B cells represent a precursor population capable of generating fully functional, conventional B lymphocytes (Figure S6A–C, Supporting Information), demonstrating that NK‐B cell‐derived B cells are phenotypically and functionally comparable to conventional B cells. These results support the conclusion that NK‐B cells from wild‐type mice represent bona fide B cell precursors rather than arrested intermediates, as they retain the capacity to differentiate into fully functional conventional B cells in a permissive environment. In contrast, the large population of NK‐B cells found in PDK1‐deficient mice reflects a developmentally arrested state. These cells fail to mature into B cells, consistent with previous reports that PDK1 signaling is essential for B cell development.^[^ ^16^, ^17^ ^]^ These findings further confirm that NK‐B cells can differentiate into B cells, rather than NK cells or NK‐B cells (Figure 5H).

We investigated whether the overexpression of PDK1 could induce the differentiation of NK‐B cells isolated from the bone marrow of PDK1f^l/fl^/Vav1‐Cre^+^ mice into either B cells or NK cells. However, PDK1‐deficient NK‐B cells were unable to survive in vitro, even when co‐cultured with SCF, IL‐3, IL‐6, or OP9 stem cells (data not shown). This suggests that PDK1 deletion led to NK‐B cell developmental arrest. Therefore, we cannot conclusively state that the NK‐B cells found in PDK1^fl/fl^/Vav1‐Cre^+^ mice are identical to those present in wild‐type mice.

Since the NK‐B cells in PDK1^fl/fl^/Vav1‐Cre^+^ mice expressed high mount of the immature marker CD117 (Figure 3I), they could potentially represent a precursor cell caused by a developmental arrest. To determine which stage of this progenitor population was affected by PDK1 deletion, we utilized two different Cre knock‐in mice models: CD122‐Cre, which mediates PDK1 deletion at the NK progenitor stage,^[^ ^20^ ^]^ and Mb1‐Cre, which mediates deletion at the large cycling pre‐B cell stage.^[^ ^15^ ^]^ We assessed the numbers of NK cells in PDK1^fl/fl^/CD122‐Cre^+^ mice and B cells in PDK1^fl/fl^/Mb1‐Cre^+^ mice. Although NK cells and B cells were significantly reduced by more than half in respective mice, no significant enrichment of NK‐B cells was observed, which is in stark contrast to the NK‐B cell enrichment seen in PDK1^fl/fl^/Vav1‐Cre^+^ mice (Figure 5I–L). Therefore, it is likely that the developmental arrest of PDK1‐deficient NK‐B cells occur prior to the pre‐B cell stage.

### 2.7 The Role of IL‐15‐E4BP4 Axis in NK‐B Cell Development

**Figure 6:**
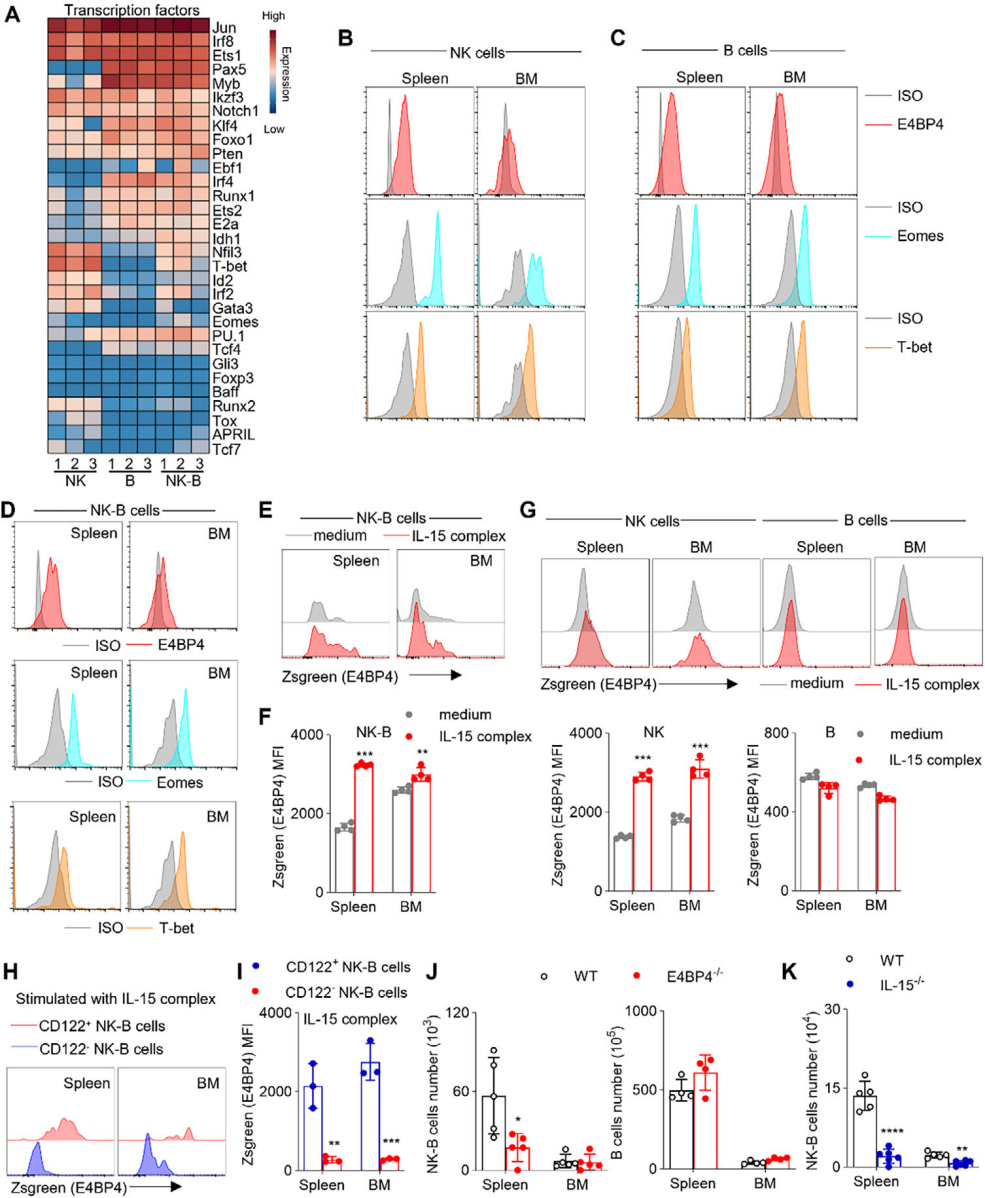
IL‐15‐E4BP4 Axis and Microbiota Regulate NK‐B Cells Existence. A) Heat map illustrating the average transcript expression of the indicated genes (n = 3). The color key indicates the expression level. The expression levels of E4BP4, Eomes and T‐bet in B) NK cells (CD3^−^CD19^−^NK1.1^+^) and C) B cells (CD3^−^CD19^+^NK1.1^−^) from resting mice spleen and bone marrow. D) The expression levels of E4BP4, Eomes and T‐bet in NK‐B cells (CD3^−^CD19^+^NK1.1^+^) from resting mice spleen and bone marrow (n = 3). The expression levels of E4BP4 in NK‐B cells (CD3^−^CD19^+^NK1.1^+^) from E4BP4 reporter mice spleen and bone marrow stimulated with IL‐15 complex E,F) the absolute MFI (Zsgreen‐MFI) were quantified (n = 4). G) The expression levels of E4BP4 in NK cells (CD3^−^CD19^−^NK1.1^+^) and B cells (CD3^−^CD19^+^NK1.1^−^) from E4BP4 reporter mice spleen and bone marrow stimulated with IL‐15 complex. The absolute MFI (Zsgreen‐MFI) were quantified (n = 4). The expression levels of E4BP4 in CD122^+^ NK‐B cells and CD122^−^ NK‐B cells from E4BP4 reporter mice spleen and bone marrow stimulated with IL‐15 complex H,I) the absolute MFI (Zsgreen‐MFI) were quantified (n = 3). J) Absolute number of NK‐B (CD3^−^CD19^+^NK1.1^+^) (n = 5) and B (CD3^−^CD19^+^NK1.1^−^) (n = 4) cells in the spleen and bone marrow of the indicated mice. K) Absolute number of NK‐B (CD3^−^CD19^+^NK1.1^+^) cells in the spleen and bone marrow of the indicated mice (n = 5). Data represent the mean ± s.d. are representative of at least three independent experiments. *p < 0.05, **p < 0.01, ***p < 0.001 and ****p < 0.0001. Unpaired Student's *t*‐tests (two‐tailed) was used to calculate these values.

According to the RNA‐Seq data, NK‐B cells showed a high expression level of B cell‐related transcription factors such as *Pax5*, *Foxo1*, and *Ebf1*. However, they also expressed NK cell‐related transcription factors including *E4bp4 (Nfil3)*, *T‐bet*, and *Eomes* (**Figure** **6**A). Since NK‐B cells are progenitor cells of B cells with NK cell markers and NK cell‐like functions, we specifically focused on the differential expression of NK cell‐related transcription factors in NK‐B cells. Under naive conditions, both B cells and NK cells express E4BP4 (Figure 6B,C). It is important to note that E4BP4 is a key transcription factor involved in NK cell development and B cell function.^[^ ^19^, ^21^ ^]^ Therefore, we investigated the expression of E4BP4 in the NK‐B cells under naive conditions (Figure 6D).

It has been reported that IL‐15 can induce the expression of E4BP4 in NK cells.^[^ ^19^ ^]^ Since the CD122 subunit of the IL‐15 receptor is also expressed on the NK‐B cells, our primary objective was to investigate whether IL‐15 could activate the expression of E4BP4 in NK‐B cells. To facilitate the observation of E4BP4 expression, we generated E4BP4 reporter mice. These mice were engineered through homologous recombination induced by CRISPR‐mediated DNA break at the 3′ end of the E4bp4 gene, resulting in the insertion of ZsGreen.^[^ ^22^, ^23^ ^]^ Upon stimulation with the IL‐15 complex, we found a significant upregulation of ZsGreen fluorescence, which represents E4BP4 expression, in both NK‐B cells and NK cells, but not in B cells (Figure 6E–G). To determine whether IL‐15‐induced E4BP4 expression in NK‐B cells depends on CD122 signaling, we administered a CD122‐blocking antibody prior to IL‐15 stimulation. This treatment significantly attenuated the upregulation of E4BP4, demonstrating that IL‐15‐mediated induction of E4BP4 is specifically dependent on CD122 signaling (Figure S7A, Supporting Information). Moreover, the IL‐15‐induced upregulation of E4BP4 expression was predominantly observed in CD122^+^ NK‐B cells (Figure 6H,I). This finding suggests that, similar to what was previously observed in NK cells, IL‐15‐induced E4BP4 expression requires CD122.

In order to genetically investigate the impact of the IL‐15‐E4BP4 axis on the existence of NK‐B cells, we utilized E4BP4^−/−^ mice, which lack NK cells. We observed a decrease in the population of NK‐B cells in the spleen while B cells were still present at normal levels (Figure 6J). Additionally, NK‐B cell numbers were moderately but significantly reduced in IL‐15^−/−^ mice, (Figure 6K). These results suggest that the IL‐15‐E4BP4 axis may have a role in the development of NK‐B cells.

### 2.8 Microbiota Regulation of NK‐B Cell Population

**Figure 7:**
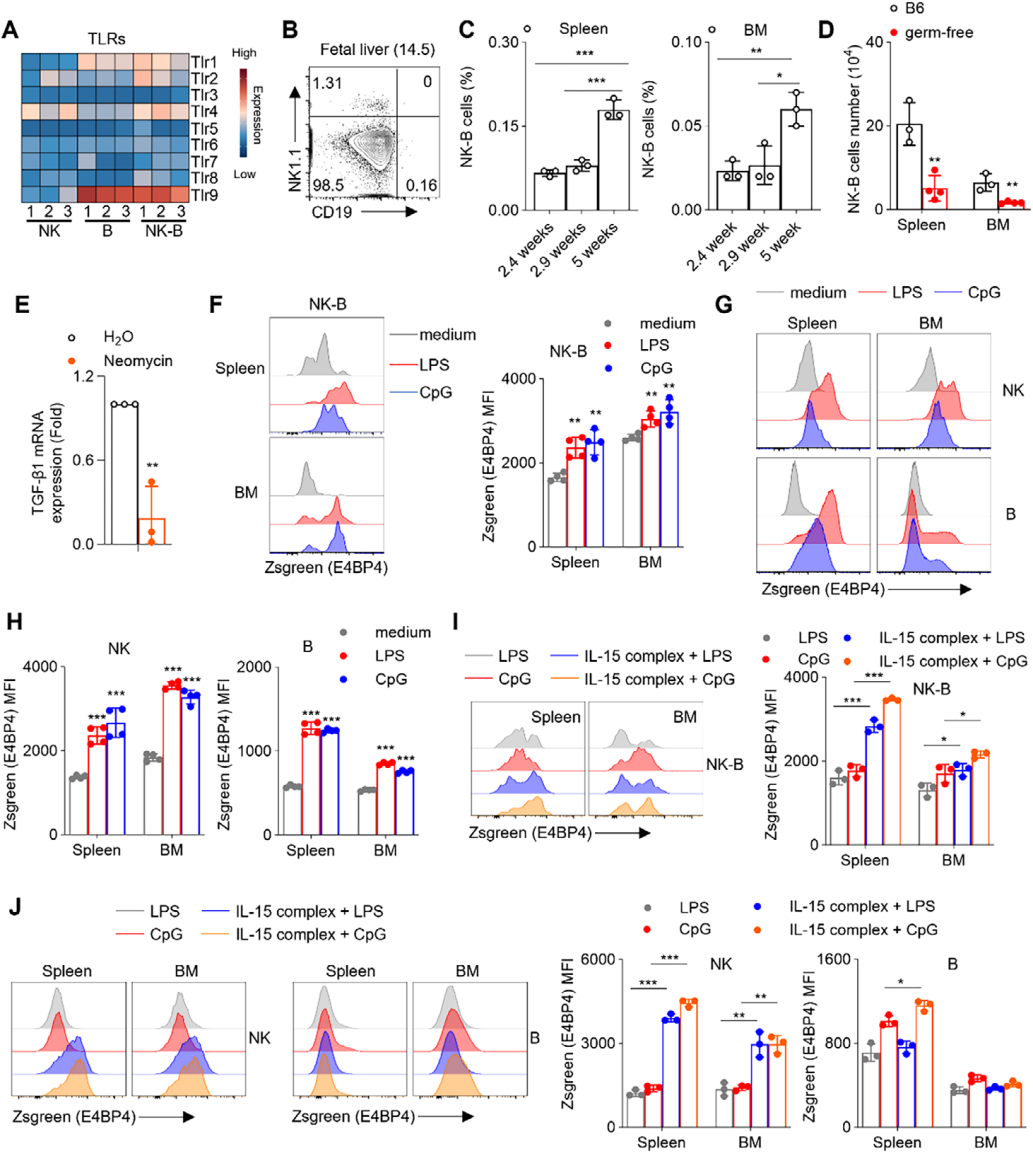
Microbiota Regulation of NK‐B Cell Population. A) Heat map illustrating the average transcript expression of the indicated genes (n = 3). The color key indicates the expression level. B) Lymphocytes in 14.5d fetal liver were isolated, stained with antibodies against CD3, 7‐AAD, CD19 and NK1.1 and analyzed by flow cytometry (n = 3). C) Lymphocytes in different ages mice spleen and bone marrow were isolated, stained with antibodies against CD3, CD19 and NK1.1 and analyzed by flow cytometry (n = 3). CD3^−^ lymphocytes were gated out for analyzing NK1.1 versus CD19 and absolute number of NK‐B cells (CD3^−^CD19^+^NK1.1^+^) were calculated (n = 3). D) Lymphocytes in germ‐free mice were isolated, stained with antibodies against CD3, CD19 and NK1.1 and analyzed by flow cytometry. CD3^−^ lymphocytes were gated out for analyzing NK1.1 versus CD19 and absolute number of NK‐B cells (CD3^−^CD19^+^NK1.1^+^) were calculated (B6, n = 3; germ‐free, n = 4). E) germ‐free mice were treated with Neomycin in water for 6 weeks and TGF‐β1 expression fold change in CD3^−^NK1.1^+^CD19^+^ NK‐B cells was analyzed using qPCR (n = 3). F) The expression levels of E4BP4 in NK‐B cells (CD3^−^CD19^+^NK1.1^+^) from E4BP4 reporter mice spleen and bone marrow stimulated with indicated stimulations (Left); the absolute MFI (Zsgreen‐MFI) were quantified (Right) (n = 4). The expression levels of E4BP4 in NK cells (CD3^−^CD19^−^NK1.1^+^) and B cells (CD3^−^CD19^+^NK1.1^−^) from E4BP4 reporter mice spleen and bone marrow stimulated with indicated stimulations G), and H) the absolute MFI (Zsgreen‐MFI) were quantified (n = 4). I) The expression levels of E4BP4 in NK‐B cells (CD3^−^CD19^+^NK1.1^+^) from E4BP4 reporter mice spleen and bone marrow stimulated with indicated stimulations, and the absolute MFI (Zsgreen‐MFI) were quantified (n = 3). J) The expression levels of E4BP4 in NK cells (CD3^−^CD19^−^NK1.1^+^) and B cells (CD3^−^CD19^+^NK1.1^−^) from E4BP4 reporter mice spleen and bone marrow stimulated with indicated stimulations (n = 3). Data represent the mean ± s.d. are representative of at least three independent experiments. *p < 0.05, **p < 0.01 and ***p < 0.001. Unpaired Student's *t*‐tests (two‐tailed) was used to calculate these values.

Analysis of our RNA‐Seq data revealed that multiple Toll‐like receptors (TLRs), including *Tlr4* and *Tlr9*, were highly expressed on NK‐B cells (**Figure** **7**A). Given that LPS stimulation could activate NK‐B cells in vivo (Figure 3H–J), it implies the potential involvement of TLR signaling in NK‐B cells. TLR4/TLR9 receptors are responsible for sensing microbiota.^[^ ^24^ ^]^ This led us to investigate the influence of the microbiome on NK‐B cell homeostasis. Since the presence of microbiota is closely associated with the birth of mice, we examined the correlation between the presence of NK‐B cells and the age of the mice. Our findings revealed that NK‐B cells were absent in the fetal liver at gestational day 14.5 (Figure 7B), but their numbers increased after birth (Figure 7C). Moreover, as mice aged, the population of NK‐B cells expanded (Figure 7C). These observations led us to speculate that microbiota plays a crucial role in the existence of NK‐B cells. To test this hypothesis, we examined NK‐B cells in germ‐free mice. We found that NK‐B cells were moderately reduced in the spleen and bone marrow of germ‐free mice compared to wild‐type mice bred in a specific pathogen‐free environment (Figure 7D). To directly examine the role of TLR9 signaling in regulating NK‐B cells, germ‐free mice were treated with a TLR9 agonist CpG. The results demonstrated that CpG administration restored the NK‐B cell population to levels comparable to those observed in wild‐type mice, indicating that TLR9 activation is sufficient to rescue NK‐B cell development under microbiota‐deficient conditions (Figure S7B, Supporting Information). Additionally, by supplying drinking water containing neomycin to wild‐type mice, we observed downregulation of TGF‐β1 mRNA expression levels in NK‐B cells (Figure 7E). These findings suggest that the microbiota is critical for NK‐B cell homeostasis and their function potentially.

Given the critical roles of E4BP4 and microbiota in the existence of NK‐B cells, we explored whether TLR4/9 signaling can regulate E4BP4 expression. We stimulated splenocytes with LPS or CpG and observed increased expression of E4BP4 in NK‐B cells, as well as B cells and NK cells (Figure 7F–H). To investigate whether TLR4/9 agonists directly regulate E4BP4 expression in NK‐B cells through a cell‐intrinsic mechanism rather than indirectly via other cell types, we stimulated sorted NK‐B cells with LPS or CpG in vitro. The results consistently demonstrated that TLR4/9 activation significantly enhanced E4BP4 expression even in the absence of other splenic or bone marrow cells (Figure S7C, Supporting Information).

Additionally, when a combination of IL‐15 with LPS/CpG was used to stimulate spleen and bone marrow cells, we observed a significant increase in ZsGreen expression in NK‐B cells compared to LPS/CpG stimulation alone (Figure 7I,J). This finding implies that TLR4/9 signaling not only induces the expression of E4BP4 itself, but also synergistically induces E4BP4 expression with IL‐15 signaling, promoting the development of NK‐B cells.

## 3 Discussion

In summary, this research uncovers a distinct population of cells called NK‐B cells that express markers of both NK cells and B cells. The NK‐B cells in this research primarily exist in the early stages of B cell development and possess unique characteristics. The study also provides insights into the regulatory mechanisms involving IL‐15, TLR4/9, and E4BP4 in maintaining the NK‐B cell population. This discovery contributes to a better understanding of the relationship between NK cells and B cells in development and immune responses, and may have implications for studying immune‐related disorders and infections Our findings demonstrate that NK‐B cells we identified in our study exhibit distinct characteristics compared to previously described NK‐B cells in mice and rhesus macaques. In mice, NK‐B cells have been reported to lack the ability to secrete IFN‐γ but can produce IL‐12 and IL‐18.^[^ ^11^ ^]^ On the other hand, in rhesus macaques, NK‐B cells capable of secreting IFN‐γ are identified as NKP46‐positive.^[^ ^13^ ^]^ Our novel NK‐B cell subset, however, differs from these previously characterized cells. These cells demonstrate the ability to produce IFN‐γ when stimulated. Additionally, the NK‐B cells we discovered exhibit a distinct surface marker profile, characterized by low or nearly no expression of NKp46. Thus, our study unveils a previously unknown population of NK‐B cells with the ability to produce IFN‐γ and a unique surface marker profile, differing from previously characterized NK‐B cells in mice and rhesus macaques. During Listeria infection, a significant expansion of the NK‐B cell population occurred, which was accompanied by a pronounced increase in the secretion of IL‐12 and IL‐18, confirming their activation upon bacterial challenge. Furthermore, the infection triggered markedly elevated expression of the effector cytokine IFN‐γ, along with the cytotoxic mediators Perforin, Granzyme A, and Granzyme B, demonstrating that NK‐B cells acquire the capacity to engage in cytotoxic immune responses during infection. These findings contribute to a deeper understanding of the heterogeneity and functional diversity of NK‐B cell subsets across different species.

Our results indicate that NK‐B cells likely arise from a distinct developmental pathway, as evidenced by their NK1.1 expression and innate immune functions, which differentiate their precursors from conventional B or NK cell lineages. The phenotypic and functional attributes of NK‐B cells are highly regulated by microenvironmental cues and activation status, the innate immune properties observed herein appear to depend on preconditioning by cytokines such as IL‐15 or microbial signals via TLR9 activation. In experimental settings lacking these signals, NK‐B cells may remain in a quiescent state and display only conventional B‐cell precursor characteristics. The precise identification of NK‐B cells in our study was accomplished using the surface marker combination CD3^−^NKp46^−^CD19⁺NK1.1⁺. Approaches that omit NK1.1 likely fail to isolate this specific subset effectively. The increased abundance of NK‐B cells in PDK1‐deficient models facilitated their detection, while the use of reporter mouse models and wild‐type NK‐B cell transfer experiments further elucidated their developmental trajectory and differentiation dynamics. Moreover, NK‐B cells may comprise functionally heterogeneous subpopulations. Our work emphasizes the NK‐B subset, which exhibits dual capacity for B‐cell differentiation and innate immune activity. Variations in surface markers, transcriptional profiles, and functional outputs among these subsets indicate that they may constitute a previously unrecognized class of immune cells.

However, this research still has many questions that need to be explored further. Through our scRNA‐seq analysis, flow cytometry analysis, and bone marrow transfer experiments, we have identified a subset of B cells called NK‐B cells that belong to the early stage of B cell development. These NK‐B cells express the NK cell marker NK1.1 and possess the ability to produce IFN‐γ. This sets them apart from conventional B cells, which primarily function in antibody production. Our data indicates that NK‐B cells exhibit low expression of CD122, suggesting that the development of NK‐B cells as progenitors of B cells may involve the loss of CD122. Furthermore, further investigation is needed to understand the developmental relationship between CD122^+^ NK‐B cells and CD122^−^ NK‐B cells. Additionally, future studies should aim to determine whether B cells derived from NK‐B cells are indistinguishable from conventional B cells.

Based on our findings, we propose that NK‐B cells constitute a unique and non‐redundant component of the innate immune arsenal against bacterial pathogens. Although conventional NK cells are well recognized for their cytotoxic functions, the adoptive transfer of NK‐B cells alone significantly restricted bacterial replication in immunodeficient hosts, demonstrating intrinsic antibacterial activity independent of NK cells. Importantly, the observed synergy‐wherein co‐transfer of NK‐B and NK cells achieved a greater reduction in bacterial burden than NK cells alone‐indicates that NK‐B cells do not play an ancillary or overlapping role. Instead, NK‐B cells appear to provide a distinct functional module that not only operates synergistically with the classical NK cell pathway but also cannot be replaced by it. Thus, NK‐B cells represent an indispensable lineage that broadens the functional architecture of innate immunity and offers a complementary defense strategy against bacterial infection.

Although NK‐B cells can produce IFN‐γ, further investigations can focus on exploring the functions and regulatory mechanisms of NK‐B cells in different diseases and infections. In addition to the functional similarities described above between NK cells and NK‐B cells, transcriptomic analysis also indicates that NK‐B cells have the capability to express effector molecules like Granzyme B and Granzyme A. However, unlike NK cells, these cells express low levels of Perforin, which is a crucial molecule for the cytotoxic function of NK cells. Therefore, it is imperative to conduct further investigations in order to identify and characterize this specific cell population, especially their in vivo functionalities. Furthermore, it should be noted that NK1.1 expression is limited to B6 mice. As a result, it is still uncertain whether a similar NK‐B cell population exists in humans. Consequently, additional research is essential to explore the potential existence of such cell populations in humans, as well as their potential applications.

The incomplete elimination of NK‐B cells in the absence of the IL‐15‐E4BP4 axis indicates the involvement of other regulatory networks, potentially mediated by other transcription factors. Further investigation into these networks is warranted to gain a better understanding of their contribution to NK‐B cell development.

## 4 Summary of the Study

1. CD3^−^ NKp46^−^CD19^+^NK1.1^+^ NK‐B cells exist in wild‐type mice, but they are extremely enriched in PDK1‐deficient mice.
2. NK‐B cells have innate cell‐like function, with capacity of IFN‐γ and TGF‐β1 secretion.
3. NK‐B cells represent B cell progenitors without the capacity to differentiate into NK cells.
4. Microbiota‐mediated TLR4/9 activation and IL‐15‐induced E4BP4 expression are crucial for the development of NK‐B cells.

## 5 Experimental Section

### Mice

Hematopoietic‐specific PDK1‐deficient mice were generated by crossing PDK1^fl/fl^ mice (a gift from D. Alessi, University of Dundee, Dundee, Scotland, UK). with Vav1‐Cre^+^ mice (B6.Cg‐Tg (Vav1‐Cre) A2Kio/J; The Jackson Laboratory).^[^ ^19^ ^]^ Rag1^−/−^γc^−/−^ mice were obtained by intercrossing *B6.129S7‐Rag1^tm1Mom/J^* (deficient in recombination‐activating gene 1) with NSG mice (*NOD.Cg‐Prkdc^scid^ Il2rg^tm1Wjl^/SzJ*; The Jackson Laboratory).^[^ ^19^ ^]^ CD122‐Cre^+^, E4BP4^−/−^, IL‐15^−/−^, IFN‐γ‐reporter (inserted eGFP before exon1 of IFN‐γ, linked with P2A) mice, R26^stop^YFP/Ncr1‐Cre^+^ reporter mice and E4BP4‐reporter mice were generated in our lab. Mb1‐Cre^+^ mice were generously provided by Dr Wanli Liu from Tsinghua University. C57BL/6J mice, CD45.1 mice, and germ‐free mice were purchased from The Jackson Laboratory. Mice used in the experiments were 6 to 10 weeks and were matched for age‐ and sex, excepted for age‐specific mouse experiments. For adoptive transfer experiments 3–5 weeks old CD45.1 mice were used. All mice were bred and kept in specific pathogen‐free animal facilities at Tsinghua University.

### Reagents

Monoclonal antibodies against mouse CD3 (#11‐0032, clone 17A2), CD19 (#12‐0193, clone eBio103), CD19 (#48‐0193, clone eBio103), NK1.1 (#17‐5941, clone PK136), B220 (#48‐0452, clone RA3‐6B2), IgM (#12‐5890, clone Eb121‐15F9), IgD (#11‐5993, clone 11–26c), NKp46 (#11‐3351, clone 29A1.4), CD122 (#48‐1222, clone TM‐b1), NKG2D (#25‐5882, clone CX5), NKG2A (#12‐5897, clone 16a11), Ly49H (#25‐5886, clone 3D10), Ly49A (#,12‐5856, clone A1), CD117 (#11‐1171, clone 2B8), CD127 (#12‐1271, clone A7R37), CD43 (#11‐0431, clone eBioR2/60), Ki‐67 (#12‐5698, clone SolA15), IFN‐γ (#12‐7311, clone XMG1.2), MHC‐I (#12‐5999, clone 28‐14‐8), E4BP4 (#12‐5927, clone S2M‐E19), Eomes (#12‐4875, clone Dan11mag), T‐bet (#53‐5825, clone eBio4B10), CD45.1 (#48‐0453, clone A20), 7‐AAD (#00‐6993), GolgiStop (#00‐4506‐51, #00‐4505‐51), and Caspase‐3 (#88‐7004) were purchased from eBioscience. Monoclonal antibody against mouse Ly49D (#555 312, clone 4E5) and Ly49C/I (#562 055, clone 5E6) were purchased from BD Biosciences. DAPI (#C1002) was purchased from Beyotime. CpG was purchased from Sangon. Ionomycin (#I0634), Phorbol 12‐myristate 13‐acetate (PMA) (#P8139), LPS (#L5293), and Neomycin (#N6386) were purchased from Sigma‐Aldrich. 15/IL‐15Ra complex was described previously.^[^ ^19^ ^]^ Mouse CD122 (TM‐beta 1, #BE0298) and IgG2b isotype control (LTF‐2, #BE0090) were purchased from BioXcell. Mouse TLR9 agonist (ODN 1585, #tlrl‐1585) and ODN control were purchased from Invivogen.

### Cells

The YAC‐1 cells (thymoma, ATCC# TIB‐160), and RMA cells (obtained from Nanjing Cobioer Biosciences) were cultured in RPMI 1640 medium (STEMCELL) supplemented with 10% fetal bovine serum (STEMCELL). For the generation of β2m‐KO RMA cells, lentivirus carrying both Cas9 nuclease and two sgRNAs targeting β2m were used. The sequences of the CRISPR sgRNAs for β2m were as follows: AGTCGTCAGCATGGCTCGCTCGG; ACTCTGGATAGCATACAGGCCGG. The lentivirus was mixed with the RMA cells, and the transduction process was conducted to introduce the genetic modifications for β2m knockout.

### Flow Cytometry

For analysis of surface markers, cells were incubated with indicated antibodies in PBS containing 2% (wt/vol) FBS. The expression level of the maker was presented as percentage or mean fluorescence intensity (MFI), which was determined by subtracting the MFI of isotype control. Intracellular staining was performed for transcription factor (E4BP4, Eomes, T‐bet) and Ki‐67. The staining procedure was carried out according to the manufacturer's instruction (#00‐5521, eBioscience). After staining, the cells were fixed with Phosflow Lyse/Fix buffer, permeabilized with Phosflow Perm buffer, and then stained with antibodies, similar to the staining procedure for Foxp3. Flow cytometry data were acquired using a Fortessa 4 Laser instrument (BD) and analyzed using FlowJo software (TreeStar).

### Immunofluorescence Assay

For in situ immunofluorescence of NK‐B cells, spleen and bone marrow cells were stained with anti‐CD3 and sorted for CD3‐negative cells by Aria 4 Laser instrument (BD). The sorted cells were then incubated with anti‐CD19 and anti‐NK1.1 antibodies for 1 h to specifically label NK‐B cells. After the staining, the cells were washed with PBS and fixed in 4% paraformaldehyde (PFA) for 30 min. Subsequently, the cells were washed three times with 0.1% Tween20/PBS and incubated with DAPI for 5 min. Following this, the cells were washed with 0.1% Tween20/PBS. Finally, the stained and fixed cells were analyzed using confocal microscopy (Olympus FV3000).

### RNA‐Sequencing

For RNA‐Seq analysis, CD3^−^CD19^+^NK1.1^+^ NK‐B cells, CD3^−^CD19^+^NK1.1^−^ B cells, and CD3^−^CD19^−^NK1.1^+^ NK cells were sorted from wild‐type mice using the BD FACSAriaII (BD) and stored in Trizol (TaKaRa, #108‐95) at ‐80 °C until library preparation. The number of sorted cells per sample ranged from 5 × 10^4^ to 2 × 10^5^. cDNA libraries were prepared using the SMART‐seq Kit V2 Pico Input RNA kit (TaKaRa, #634 418) following the manufacturer's instructions. The samples were then submitted to the BioMicro center at Anoroad company for library construction and sequencing. Paired‐end reads data were assessed for quality using the MultiQC tool (https://multiqc.info/) based on typical RNA‐Seq experiments. Strand‐specific cDNA libraries were constructed and subjected to high‐throughput sequencing on an Illumina NovaSeq 6000 platform, generating 150 bp paired‐end reads. A minimum sequencing depth of 40 million raw reads per sample (≈12 Gb) was achieved to ensure robust detection of both medium‐ and low‐abundance transcripts. The GC content was examined to ensure a single peak and check adapter content. The RNA‐Seq reads were then mapped to the genome (mm10) using TopHat. The resulting alignment provided the number of reads mapped to each feature. FeatureCounts was used to retrieve read counts mapping to exons, which were subsequently summarized at the gene level. Once the featureCount matrix was generated, further analysis including Principal Components Analysis (PCA), Heatmap, and Gene Ontology (GO) analysis was performed using R programming. Differential gene expression analysis was conducted using DEseq2 (Version 1.28.1). Genes with a *p*‐value < 0.05 and a log_2_ fold change (log_2_FC) > 1 or log_2_FC < ‐1 were considered as differentially expressed.

### Adoptive Cell Transfer

A mixture of 2 × 10^5^ CD3^−^CD19^+^NK1.1^+^ NK‐B cells were sorted from the bone marrow of CD45.1 mice using the BD FACSAriaII (BD) was intravenously transferred into Rag1^−/−^γc^−/−^ mice. At 8 weeks after transplantation, flow cytometry analysis was performed to examine the presence of donor‐derived cells (CD45.1^+^) in the spleen and bone marrow of recipient mice.

### In Vitro NK Cell Function Assay

To assess the production of IFN‐γ in NK cells, B cells, and NK‐B cells, splenocytes (2 × 10^6^) from PolyI:C‐treated mice were co‐cultured with PMA (50 ng mL^−1^) plus ionomycin (1 mM), or equal numbers of the respective target cells for 5 h in the presence of GolgiStop (eBioscience). After the co‐culture, cells were stained with antibodies against CD3, CD19, and NK1.1 to identify the specific cell populations. Next, the cells were fixed and permeabilized using Cytofix/Cytoperm Buffer (eBioscience) and subsequently stained with IFN‐γ antibody.

### Listeria Infection, LPS Injection and Specific Water

Regarding the Listeria infection model, mice were intravenously injected with 1 × 10^5^ colony‐forming units (CFU) of Listeria. After 7 days, the mice were euthanized for NK‐B cells qPCR experiments to analyze gene expression levels. The control group received an injection of phosphate‐buffered saline (PBS). In the LPS model, mice were intraperitoneally injected with 5 mg kg^−1^ of lipopolysaccharide (LPS). After 4 days, the mice were euthanized for NK‐B cells qPCR experiments to analyze gene expression levels and IFN‐γ‐reporter mice experiments. The control group received an injection of PBS. For the specific‐water model, mice were fed with water containing 5 mg mL^−1^ of Neomycin. After 6 weeks, the mice were euthanized for NK‐B cells qPCR experiments to analyze gene expression levels. The control group received normal water.

### Real‐Time Polymerase Chain Reaction

RNA was extracted from indicated cells using Trizol reagent (TaKaRa, #108‐95), followed by reverse transcription into cDNAs using oligo d(T)n primers (Vazyme, #R111). Real‐time PCR (Vazyme, #Q311) was conducted using a Bio‐Rad CFX96 Real‐Time System with primers specific to the target genes. The expression levels of the genes were quantified relative to the expression of β‐actin after normalization. vpreb primer sequences were ATGCTGCTGGCCTATCTCACAGG (forward) and ATGGTCGTTGCTCAGGGTACAGG (reverse). λ5 primer sequences were GCGGAATTCTCAGCAGAAAGGAGCAGAGCT (forward) and GCGAAGCTTACACACTACGTGTGGCCTTGT (reverse). Iga primer sequences were CCTGCCTCTCCTCCTCTTCTTGTC (forward) and GACTGAAGGCTGAACCACCATGTG (reverse). Igb primer sequences were GTGCCCATCTTCCTGCTACTTGAC (forward) and TGCTCTCCTACCGACCACTTTACC (reverse). V_k_‐JC_K_ primer sequences were GGCTGCAGCTTCAGTGGCAGTGGATCAGGAAC (forward) and TTGGTCAACGTGAGGGTGCTG (reverse). V_H_7183 primer sequences were CGGTACCAAGAACAACCTGTACCTGCAAATGACC (forward) and ATGCAGATCTCTGTTTTTGCCTCC (reverse). V_H_J558 primer sequences were CGAGCTCTCCAACACAGCCTACATGCAACTCAAC (forward) and ATGCAGATCTCTGTTTTTGCCTCC (reverse). TGF‐β1 primer sequences were CACAGAGAAGAACTGCTGTG (forward) and AGGAGCGCACAATCATGTTG (reverse). b‐actin primer sequences were GTGACGTTGACATCCGTAAAGA (forward) and GCCGGACTCATCGTACTCC (reverse).

### Detection of E4BP4

To detect the reactivity of E4BP4 in NK‐B cells, NK cells, and B cells, 2 × 10^6^ splenocytes and bone marrow cells were incubated in 24‐well plates with various stimuli. The stimuli included mIL‐15/IL‐15Rα complex (10 ng mL^−1^), LPS (5 µg mL^−1^), and CpG (2 µg mL^−1^). After 18–24 h, the cells were collected for analysis by flow cytometry. Cells treated with medium alone were used as control samples.

### Gene‐Expression Analysis

The scRNA‐seq dataset of wild‐type mice CD3^−^NK1.1^+^ spleen cells in Figure 3C–G were obtained from the Gene Expression Omnibus (GEO; GSE249425)^[^ ^25^ ^]^ and then processed using the R package Seurat.

### Statistical Analysis

The statistical analysis was performed using Prism 7 software. Unpaired Student's *t*‐tests (two‐tailed) were conducted to determine the significance. A *p*‐value less than 0.05 was considered as statistically significant. The significance levels were denoted as *p < 0.05, **p < 0.01, and ***p < 0.001. The data were presented as the mean ± standard deviation (SD).

### Ethical Approval

All mouse experiments were approved by the Animal Ethics Committee of Tsinghua University (18‐DZJ‐1), Beijing, China

## Conflict of Interest

The authors declare no conflict of interest.

## Author Contributions

J.H. and X.H. contributed equally to this work. J.H. and X.H. conceived the project, designed the experiments, and performed them. X.F., Y.D., M.R., S.D., X.Y., and D.C. analyzed the data. L.Z. provided the reporter mice. S.C. and M.Y. revised the manuscript. Z.D. designed the study, supervised the research, revised the manuscript, and approved the published version of the manuscript.

## Supporting information



Supporting Information

## Data Availability

The previously published scRNA‐seq data of GSE249425 (https://www.ncbi.nlm.nih.gov/geo/query/acc.cgi?&acc¼GSE249425) from the Gene Expression Omnibus database were used in this paper.^[^ ^25^ ^]^ NK cells from three independent samples were pooled together for single‐cell RNA sequencing. We analyzed previously published single‐cell RNA‐seq datasets comprising 66613 cells from human bone marrow and 200664 cells from spleen, which were obtained from the CellxGene portal (https://cellxgene.cziscience.com/). The remaining data can be found in the article and its supplementary data files or can be obtained from the corresponding authors upon request.
