# Supplementary material for: A Microbiota‐ and IL‐15‐Dependent Innate‐Like B Cell Progenitor Expressing E4BP4
Source: Adv Sci (Weinh). 2025 Dec 2;13(8):e12444. doi: 10.1002/advs.202512444 (PMC12884798; doi:10.1002/advs.202512444)
Supplement: Supplementary file 1 — Supporting Information [file ADVS-13-e12444-s001.doc]

Supporting information

**A Microbiota- and IL-15-Dependent Innate-like B Cell Progenitor Expressing E4BP4**

Junming He*, Xinlei Hou, Xiaomei Feng, Yayun Dong, Mengqi Ren, Surong Deng, Xinru Yang, Donglin Chen, Lingna Zhao, Shasha Chen*, Meixiang Yang* and Zhongjun Dong*

X. Feng, Y. Dong, M. Ren, S. Deng, X. Yang, S. Chen, Z. Dong

Department of Allergy, the First Affiliated Hospital of Anhui Medical University and Institute of Clinical Immunology, Anhui Medical University, Hefei, 230032, China. Innovative Institute of Tumor Immunity and Medicine (ITIM), Hefei, 230032, China. Anhui Province Key Laboratory of Tumor Immune Microenvironment and Immunotherapy, Hefei, 230032, China. Inflammation and Immune Mediated Diseases Laboratory of Anhui Province, Anhui Medical University, Hefei, 230032, China

E-mail: chenshasha.26@163.com

Lead Contact E-mail: dongzj@mail.tsinghua.edu.cn

X. Hou, D. Chen, L. Zhao, Z. Dong

State Key Laboratory of Membrane Biology, School of Medicine and Institute for Immunology, Tsinghua University, Beijing, 100084, China

Lead Contact E-mail: dongzj@mail.tsinghua.edu.cn

M. Yang

The Biomedical Translational Research Institute, Guangzhou Key Laboratory for Germ-Free Animals and Microbiota Application, Key Laboratory of Ministry of Education for Viral Pathogenesis & Infection Prevention and Control, School of Medicine, Jinan University, Guangzhou, 510632, China

E-mail: mxyang@jnu.edu.cn

J. He

Beijing Tsinghua Changgung Hospital, School of Clinical Medicine, Tsinghua University, Beijing 102218, China. Institute for Organ Transplant and Bionic Medicine, Tsinghua University, Beijing 102218, China

E-mail: hejunming137@163.com


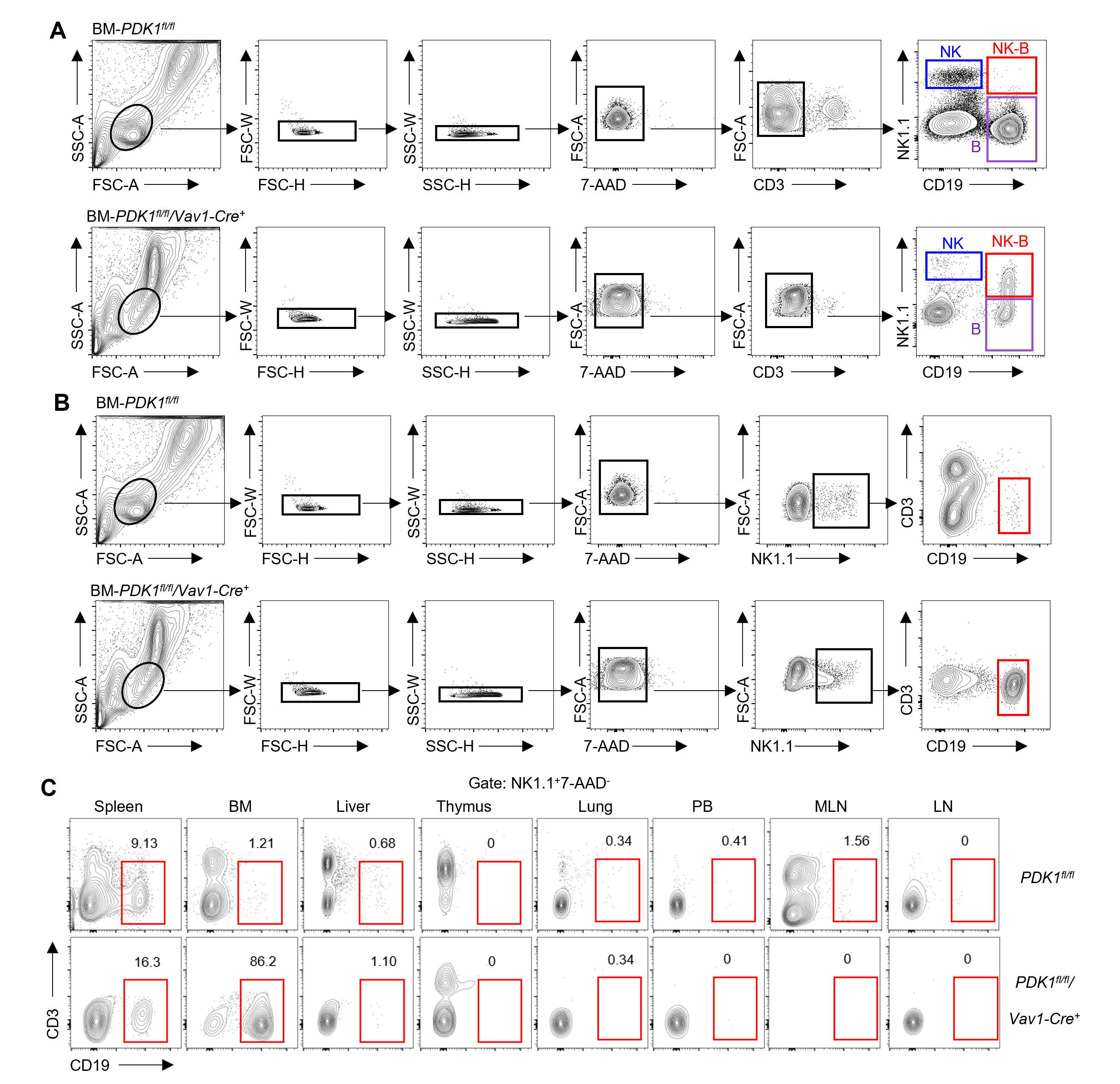


**Figure S1.** NK-B Gating Strategy. A-B) Two different gating strategy of CD3-7-AAD^-^NK1.1^+^CD19^+^ cells in mice. C) Lymphocytes in different mouse tissues were isolated, stained with antibodies against CD3, CD19 and NK1.1 and analyzed by flow cytometry. Dead cells were excluded by 7-AAD staining. CD3^-^NK1.1^+^ lymphocytes were gated out for analyzing CD3 versus CD19.


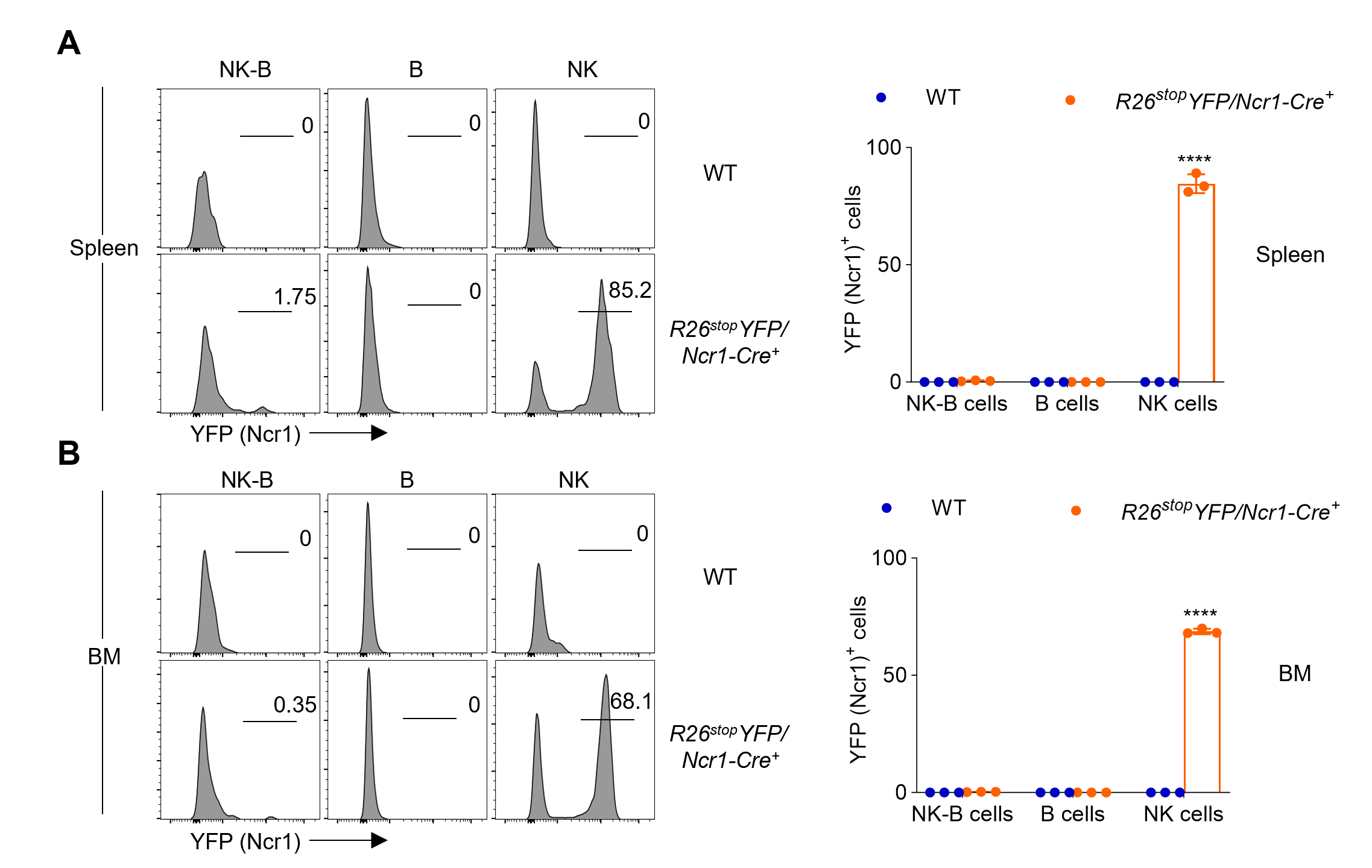


**Figure S2.** Fate-mapping Analysis of NKp46 Expression in NK cells, B cells, and NK-B cells. A-B) Flow cytometric analysis of YFP (*Ncr1*) expression in NK cells, B cells, and NK-B cells from the spleen and bone marrow of R26^stop^YFP/Ncr1-Cre^+^ reporter mice (n=3). Data represent the mean ± s.d. are representative of at least three independent experiments. *P < 0.05, **P < 0.01, ***P < 0.001 and ****P < 0.0001. Unpaired Student’s t-tests (two-tailed) was used to calculate these values.


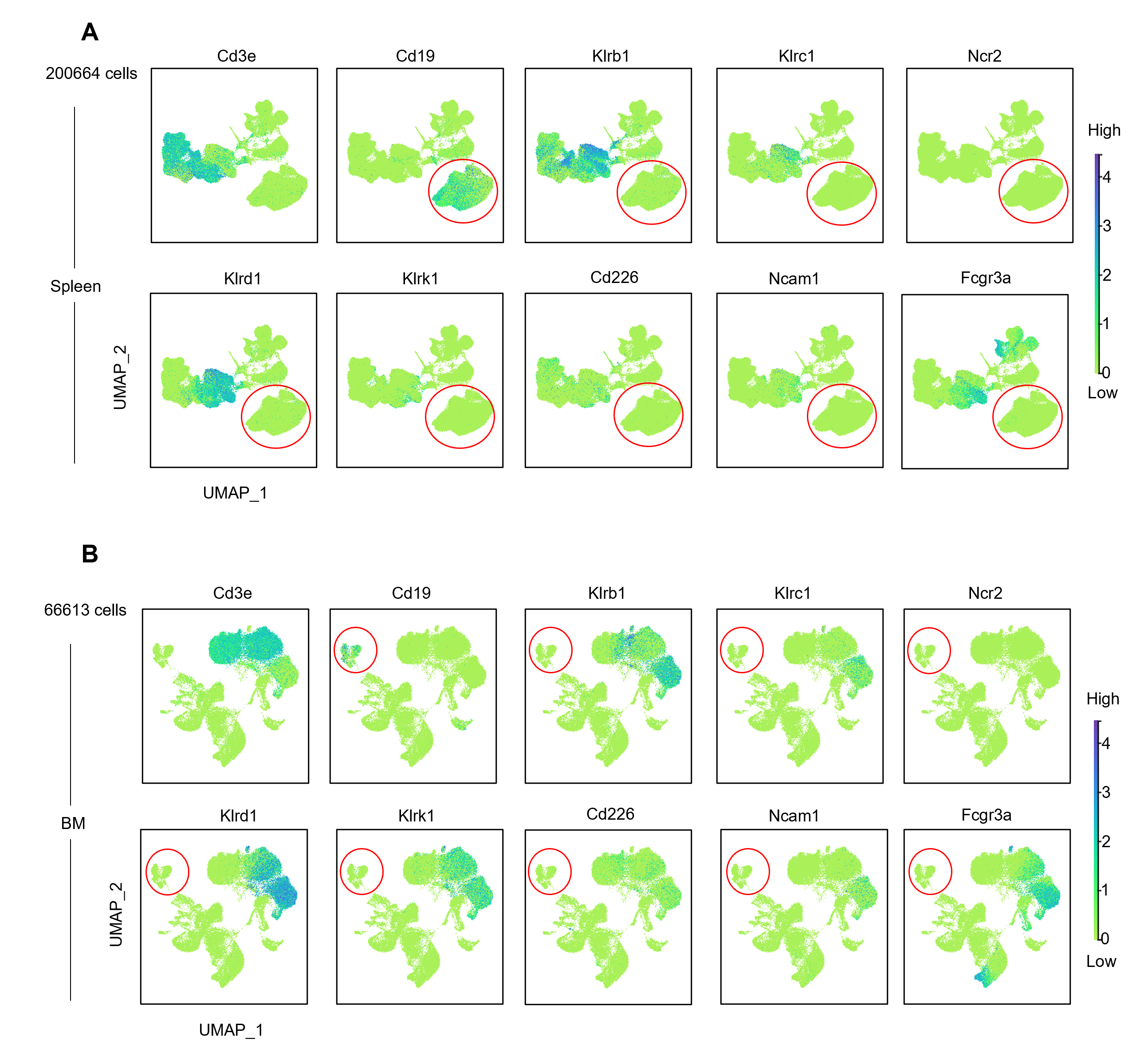


**Figure S3.** NK1.1 Homolog Expression in CD3⁻NKp46⁻CD19⁺ Cells. A–B) UMAP visualization of human splenic (top) and bone marrow (bottom) cells was generated using published single-cell RNA-seq datasets (https://cellxgene.cziscience.com/). CD3⁻NKp46⁻CD19⁺ cells expressing homologs of NK1.1 were analyzed in both human spleen and bone marrow. Expression patterns of key human NK cell-associated receptors are visualized on the UMAP embeddings, with color intensity corresponding to gene expression levels.

**
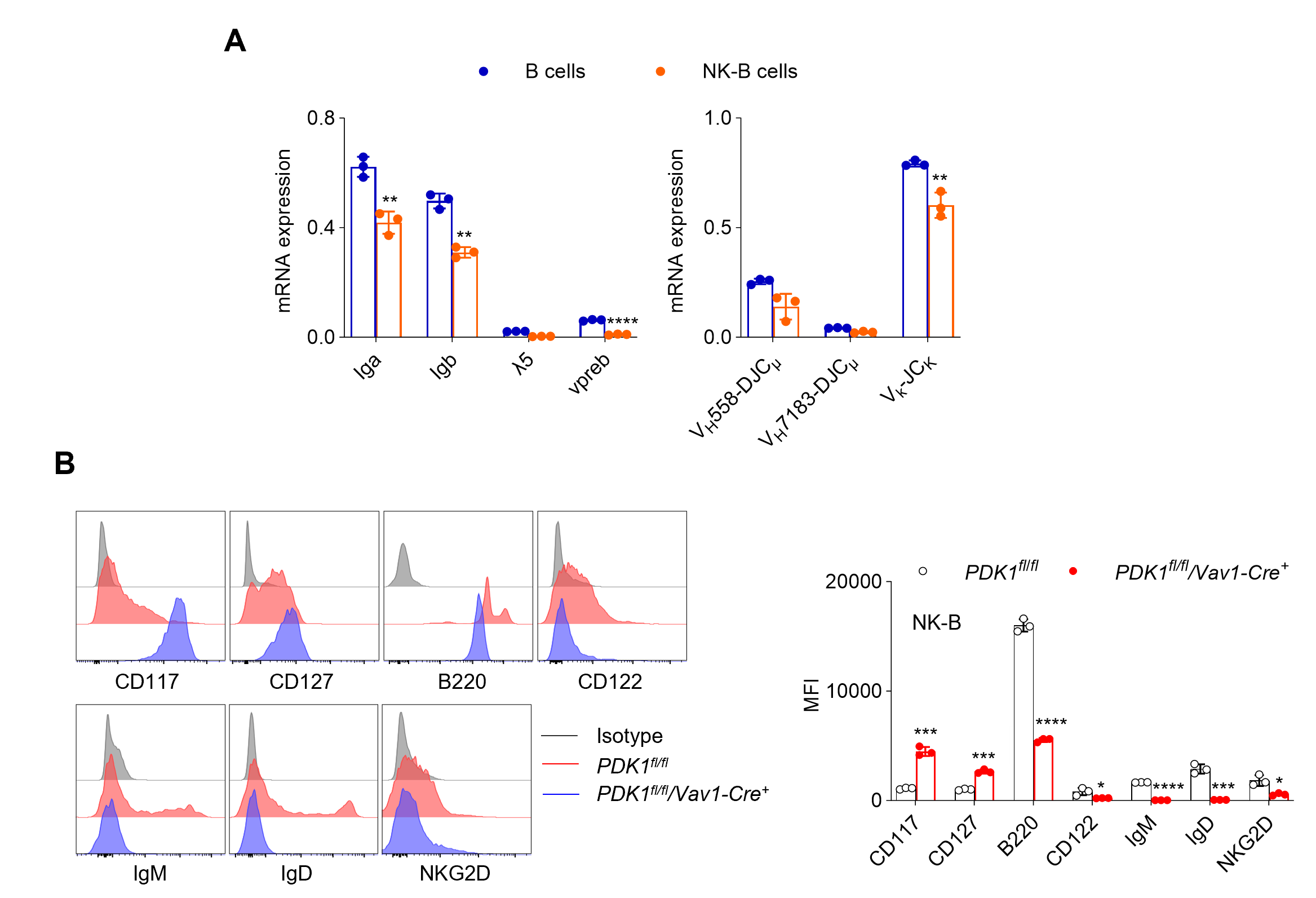
**

**Figure S4.** NK-B Cells Have Unique Signature Features. A) pre-BCR component mRNA expression, V-DJ mRNA expression as well as the expression of the distal (V_H_J588), proximal (V_H_J7183) V_H_ and V_k_-J_k_ regions germline transcripts in CD3^-^NK1.1^+^CD19^+^ NK-B cells and CD3^-^NK1.1^-^CD19^+^ B cells from wild-type mice bone marrow was analyzed using qPCR (n=3). B) Flow cytometry analysis of expression of the indicated receptors on bone marrow CD3^-^NK1.1^+^CD19^+^ NK-B cells from PDK1^fl/fl^ mice (red line) and PDK1^fl/fl^/Vav1-Cre^+^ mice (blue line) (n=3). Data represent are representative of at least three pairs of mice. Data represent the mean ± s.d. are representative of at least three independent experiments. *P < 0.05, **P < 0.01, ***P < 0.001 and ****P < 0.0001. Unpaired Student’s t-tests (two-tailed) was used to calculate these values.


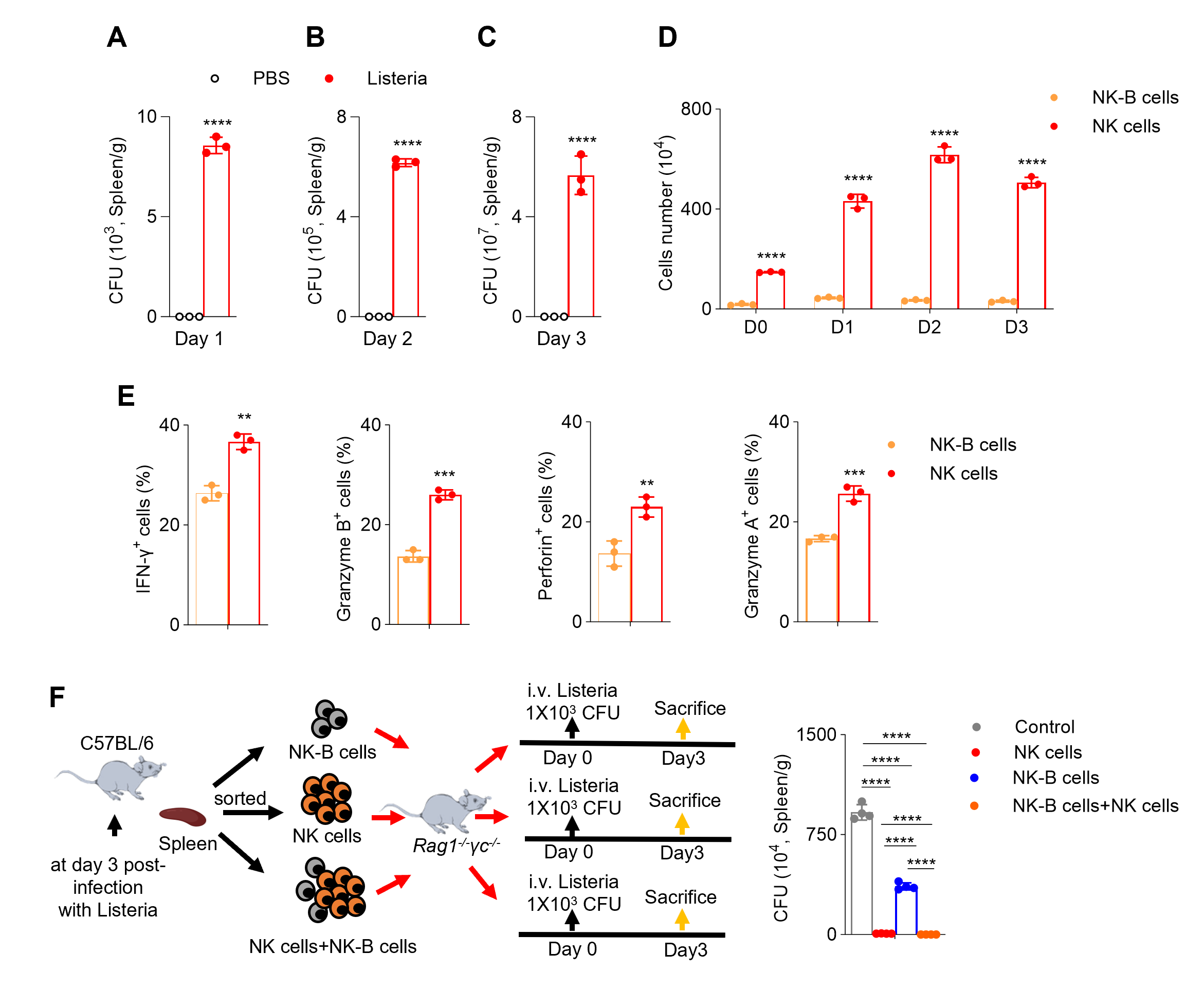


**Figure S5.** NK-B cells exhibit a distinct activation profile and contribute to the control of Listeria infection. A-C) Measurement of bacterial load in the spleen of wild-type mice at different time points following intravenous infection with 1×10⁵ CFU of Listeria (n=3). D) Wild-type mice were infected with Listeria and analyzed at various time points. The percentages of NK-B cells (CD3^-^CD19^+^NK1.1^+^) and NK cells (CD3^-^CD19^-^NK1.1^+^) were determined (n=3). E) Wild-type mice were infected with Listeria and sacrificed at the indicated time points. Spleens were harvested, and the percentages of IFN-γ^+^, Perforin^+^, Granzyme A^+^, and Granzyme B^+^ within gated NK-B cells (CD3^-^CD19^+^NK1.1^+^) and NK cells (CD3^-^CD19^-^NK1.1^+^) were analyzed (n=3). F) First, donor NK and NK-B cells were purified from wild-type mice at day 3 post-infection with 1×10⁵ CFU of Listeria for adoptive transfer. These pre-activated cells were used to reconstitute Rag1⁻/⁻γc⁻/⁻ recipients in the following groups: NK cells alone, NK-B cells alone, or both at an 18:1 ratio; an unreconstituted group was included as a control. Finally, all mice were challenged intravenously with 1000 CFU of Listeria, and the splenic bacterial load was quantified at the indicated time points (n=4). Data represent the mean ± s.d. are representative of at least three independent experiments. *P < 0.05, **P < 0.01, ***P < 0.001 and ****P < 0.0001. Unpaired Student’s t-tests (two-tailed) was used to calculate these values.


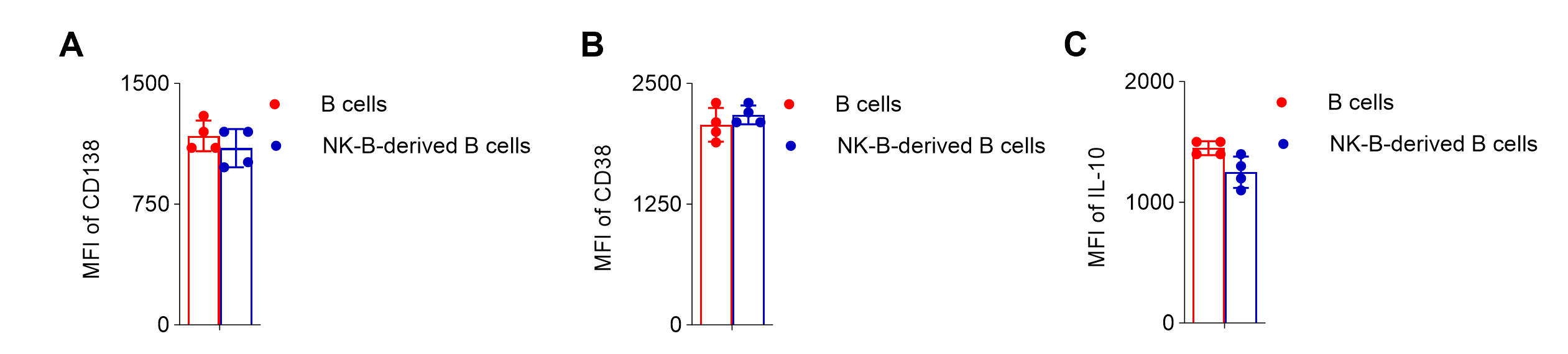


**Figure S6.** Functional Equivalence of NK-B Cell-Derived B Cells and Conventional B Cells. CD3^-^NK1.1^+^CD19^+^ NK-B cells from CD45.1^+^ mice bone marrow were sorted and transferred into CD45.2^+^ Rag1^-/-^γc^-/-^ mice and analyzed at 2 months. The differentiation of transferred NK-B cells (CD3^-^CD19^+^NK1.1^+^) into B cells was assessed by flow cytometry. Expression of CD38 A), CD138 B), and IL-10 C) was compared between these cells and wild-type B cells (n=4). Data represent the mean ± s.d. are representative of at least three independent experiments. *P < 0.05, **P < 0.01 and ***P < 0.001. Unpaired Student’s t-tests (two-tailed) was used to calculate these values.


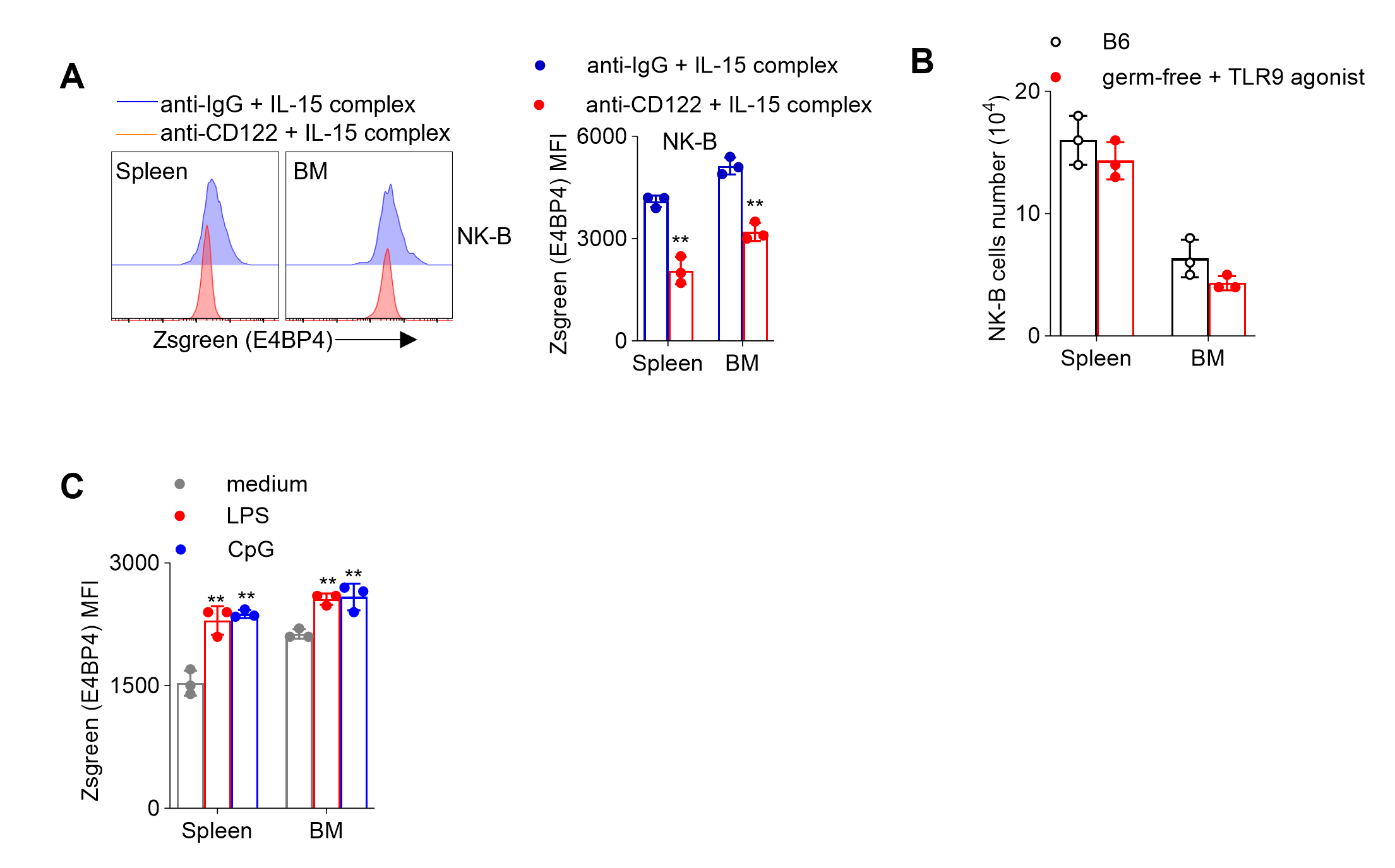


**Figure S7.** Dependence of NK-B Cells on IL-15 and Microbiota/TLR Signaling Pathways. A) E4BP4 reporter mice were intraperitoneally administered an anti-CD122 blocking antibody at a dose of 200μg per mouse every other day for two weeks. The expression levels of E4BP4 in NK-B cells (CD3^-^CD19^+^NK1.1^+^) from E4BP4 reporter mice spleen and bone marrow stimulated with IL-15 complex, and the absolute MFI (Zsgreen-MFI) were quantified (n=3). B) Germ-free mice were intravenously injected with 20μg of TLR9 aganist per mouse every other day for a duration of two weeks, stained with antibodies against CD3, CD19 and NK1.1 and analyzed by flow cytometry. CD3^-^ lymphocytes were gated out for analyzing NK1.1 versus CD19 and absolute number of NK-B cells (CD3^-^CD19^+^NK1.1^+^) were calculated (n=3). C) The expression levels of E4BP4 in sorted NK-B cells from E4BP4 reporter mice spleen and bone marrow stimulated with LPS and CPG; the absolute MFI (Zsgreen-MFI) were quantified (n=3). Data represent the mean ± s.d. are representative of at least three independent experiments. *P < 0.05, **P < 0.01 and ***P < 0.001. Unpaired Student’s t-tests (two-tailed) was used to calculate these values.
